# Deep learning-based behavioral analysis reaches human accuracy and is capable of outperforming commercial solutions

**DOI:** 10.1038/s41386-020-0776-y

**Published:** 2020-07-25

**Authors:** Oliver Sturman, Lukas von Ziegler, Christa Schläppi, Furkan Akyol, Mattia Privitera, Daria Slominski, Christina Grimm, Laetitia Thieren, Valerio Zerbi, Benjamin Grewe, Johannes Bohacek

**Affiliations:** 1 Laboratory of Molecular and Behavioral Neuroscience, Institute for Neuroscience, Department of Health Sciences and Technology, ETH Zurich, Zurich, Switzerland; 2 Neuroscience Center Zurich, ETH Zurich and University of Zurich, Zurich, Switzerland; 3 Neural Control of Movement Lab, Department of Health Sciences and Technology, ETH Zurich, Zurich, Switzerland; 4 Experimental Imaging and Neuroenergetics, Institute of Pharmacology and Toxicology, University of Zurich, Zurich, Switzerland; 5 Institute of Neuroinformatics, University of Zurich and ETH Zurich, Zurich, Switzerland; 6 Department of Information Technology and Electrical Engineering, ETH Zurich, Zurich, Switzerland

**Keywords:** Anxiety, Behavioural methods

## Abstract

To study brain function, preclinical research heavily relies on animal monitoring and the subsequent analyses of behavior. Commercial platforms have enabled semi high-throughput behavioral analyses by automating animal tracking, yet they poorly recognize ethologically relevant behaviors and lack the flexibility to be employed in variable testing environments. Critical advances based on deep-learning and machine vision over the last couple of years now enable markerless tracking of individual body parts of freely moving rodents with high precision. Here, we compare the performance of commercially available platforms (EthoVision XT14, Noldus; TSE Multi-Conditioning System, TSE Systems) to cross-verified human annotation. We provide a set of videos—carefully annotated by several human raters—of three widely used behavioral tests (open field test, elevated plus maze, forced swim test). Using these data, we then deployed the pose estimation software DeepLabCut to extract skeletal mouse representations. Using simple post-analyses, we were able to track animals based on their skeletal representation in a range of classic behavioral tests at similar or greater accuracy than commercial behavioral tracking systems. We then developed supervised machine learning classifiers that integrate the skeletal representation with the manual annotations. This new combined approach allows us to score ethologically relevant behaviors with similar accuracy to humans, the current gold standard, while outperforming commercial solutions. Finally, we show that the resulting machine learning approach eliminates variation both within and between human annotators. In summary, our approach helps to improve the quality and accuracy of behavioral data, while outperforming commercial systems at a fraction of the cost.

## Introduction

Accurate analysis of rodent behavior is crucial when assessing treatment efficacy in preclinical research. The rapid development of new tools and molecular interventions in rodents, as well as the growing number of available transgenic mouse lines, increase the need to accurately and efficiently detect and quantify rodent behavior [1, 2]. Typically, behavioral analysis relies on commercial equipment to track an animal’s path of movement or measure the time spent in specific areas of testing arenas. Commercial solutions usually use video tracking or infrared beam grids, and are available either as stand-alone software packages (EthoVision, Anymaze), or are integrated with hardware to create all-in-one behavioral analysis apparati (e.g., TSE Systems, Campden Instruments, Med Associates). Such systems enable researchers to conduct semi high-throughput behavioral screenings [3]. However, commercial solutions are not only expensive, but also lack the ability to flexibly define and score specific behaviors of interest and often cannot be adapted to fit changing experimental needs. Even more problematically, their tracking ability is often suboptimal and they measure ethological behaviors with poor sensitivity [4–7]. As a result, human scoring has remained the gold standard when quantifying ethological behaviors. However, human annotators tire when performing repetitive tasks and their performance may vary across days. Further, the complexity of animal behavior can overwhelm the annotator, and subtle differences in the definition of complex behaviors can further increase the variability between human annotators, leading to high inter-rater variability [4, 8–11].

Recently, major advances in machine learning have given rise to the first descriptions of unsupervised analyses of behavior, revealing the stunning temporal and structural complexity of rodent behavior [12–16]. However, these advanced analyses are challenging for many biology and behavioral research labs to establish, which probably explains why they have not yet been widely implemented by the behavioral research community. An elegant and accessible implementation of deep learning for motion tracking and markerless pose estimation is DeepLabCut (DLC), an open source software package that has been rapidly disseminating across animal behavior laboratories throughout the world [17, 18]. In contrast to commercial systems, DLC allows the user to define and track specific points of interest (e.g. specific body parts). Due to this increased level of detail and flexibility, we tested if DLC could be harnessed to replace existing commercial tracking packages, and whether it could be combined with machine learning to help reach human accuracy when scoring complex, ethological behaviors. Behavior tracking and analysis is performed in a vast number of behavioral tests for rodents. In this report, we focus on three of the most popular behavioral assays routinely used in preclinical research: the open field test [19]; the elevated plus maze [20, 21]; and the forced swim test (FST) [22]. A search on pubmed showed that these tests have been used in more than 10,000 research papers to date, with a steady increase over the last decade (Fig. S1). Several task-specific ethological behaviors have been documented [23, 24] including head dipping in the elevated plus maze [21, 25]; rearing in the open field test [6, 26, 27]; and floating in the FST [28], which are three prominent examples of ethological behaviors associated with emotional and disease states [29, 30]. For instance, reduced exploration (rearing/head dipping) indicates anxiety [6], and floating in the FST has been linked to adaptive stress-coping behaviors [31], although it is also frequently used to screen the antidepressant activity of new drugs [32]. Therefore, being able to accurately score and report these behaviors adds an important layer of information to the basic motion path of the animal. In this work we couple DLC-tracking with supervised machine learning. We then carefully compare this approach to commercial platforms (the video tracking software EthoVision XT14 from Noldus, and the ‘all-in-one’ Multi Conditioning System from TSE systems), and to behavior rated by several human annotators (the gold standard).

## Materials and methods

A detailed description of all procedures is found in Supplementary Materials and methods.

### Animals

C57BL/6J (C57BL/6JRj) mice (male, 2.5 months of age) were obtained from Janvier (France). Mice were maintained in a temperature- and humidity-controlled facility on a 12-h reversed light–dark cycle (lights on at 08:15 am) with food and water ad libitum. Mice were housed in groups of 5 per cage and used for experiments when 2.5–4 months old. For each experiment, mice of the same age were used in all experimental groups to rule out confounding effects of age. All tests were conducted during the animals’ active (dark) phase from 12–5 pm. Mice were single housed 24 h before behavioral testing in order to standardize their environment and avoid disturbing cage mates during testing [33, 34]. All procedures were carried out in accordance to Swiss cantonal regulations for animal experimentation and were approved under license 155/2015.

### Open field test (OFT)

OFT took place inside sound insulated, ventilated multi conditioning chambers (TSE Systems Ltd, Germany). The open field arena (45 × 45 × 40 cm [*L* × *W* × *H*]) consisted of four transparent Plexiglas walls and a light gray PVC floor. Animals were tested for 10 min under dim lighting (4 lux). Distance, time in center, supported rears and unsupported rears were recorded.

### Elevated plus maze (EPM)

The EPM was made from gray PVC, with arms measuring 65.5 × 5.5 cm (*L* × *W*), elevated 61.5 cm. Light intensity in the open arms was at 19–21 lux. All EPM tests were 10 min in duration. Distance, velocity, time in zone (open/closed arms + center) and head dips were recorded.

### Forced swim test (FST)

Animals were forced to swim in a plastic beaker (20 cm diameter, 25 cm deep) filled to 17 cm with 17.9–18.1 °C water for 6 min.

### Noldus EthoVision

EthoVision XT14 was used to acquire all forced swim and elevated plus maze videos and to analyze all of the open field videos. The automatic animal detection settings were used for all tests, slight tuning of these settings was performed using the fine-tuning slider in the automated animal detection settings to ensure the animals could be tracked throughout the entire arena. We ensured that there was a smooth tracking curve and that the centerpoint of the animal remained stable before analysis took place.

### DeepLabCut (DLC)

**Fig. 1:**
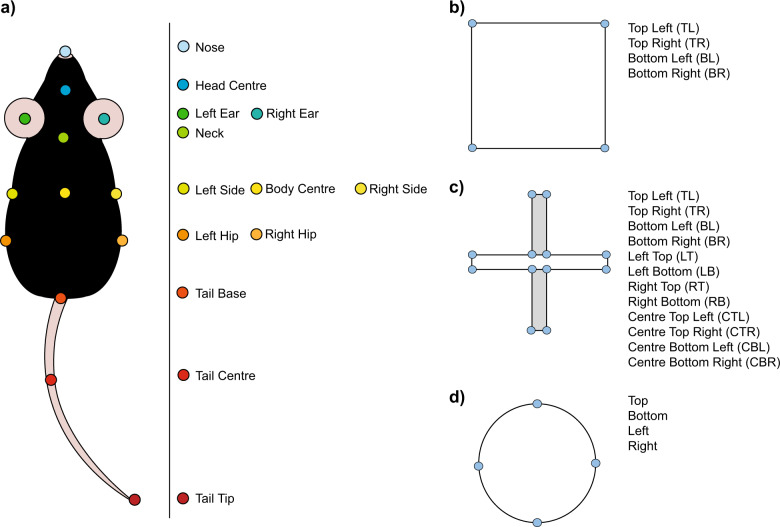
The labels used to train the DLC networks. **a** The standardized points of interest used to track the animal. The points of interest required to track the animal in the open field (**b**), the elevated plus maze (**c**) and the forced swim test (**d**).

DeepLabCut 2.0.7 was used to track 13 body points and several points of the various arenas (Fig. 1). The networks for different tests were trained using 10–20 frames from multiple randomly selected videos for 250,000–1,030,000 iterations (for details see Supplementary Materials and methods). The data generated by DeepLabCut were processed using custom R Scripts that are available online (https://github.com/ETHZ-INS/DLCAnalyzer).

### TSE Multi Conditioning System

Locomotion was tracked using an infrared beam grid; an additional beam grid was raised 6.5 cm above the locomotion grid to measure rearing. The central 50% (1012.5 cm^2^) was defined as the center of the arena. To automatically distinguish supported from unsupported rears, we empirically determined the area in which mice could not perform a supported rear. Thus, all rears within 12.5 cm of the walls were considered supported rears, while rears in the rest of the field were considered unsupported rears. Rearing was defined as an interruption of a beam in the *z*-axis for a minimum of 150 ms. If another rear was reported within 150 ms of the initial rear, it was counted as part of the initial rear.

### Analysis of DLC coordinates

X and Y coordinates of DLC-tracking data were imported into R Studio (v 3.6.1) and processed with custom scripts (https://github.com/ETHZ-INS/DLCAnalyzer). Values of points with low likelihood (>0.95) were removed and interpolated using the R package “imputeTS” (v 2.7). The speed and acceleration of each point was determined by integrating the animal’s position over time. Points of interest relating to the arenas were tracked and median XY coordinates were used to define the arenas in silico. The pixel-to-cm conversion ratio for each video was determined by comparing the volume of the arena in silico in px^2^ to the measured size of the arena in cm^2^. Zones of interest were calculated from the arena definitions using polygon-scaling functions. Detailed descriptions of how individual behaviors were computed can be found in Supplementary Materials and methods.

### Time resolved skeleton representation

A position and orientation invariant skeletal representation was created from the DLC tracked coordinates at each frame. Based on distances, angles and areas, 22 variables were used as features for the supervised machine learning. For details of the feature description see Supplementary Materials and methods.

### Machine learning approach

In order to create a training dataset, 20 videos of the OFT were manually labeled (using VIA video annotator [35]), indicating the onset and offset of selected behaviors. Labeled behaviors include ‘supported rear’, ‘unsupported rear’, and by default ‘none’. Videos were labeled by three independent raters. These sets of labeling data were used to train multiple neuronal networks for the classification of the selected behaviors. Labeling data are deposited online (https://github.com/ETHZ-INS/DLCAnalyzer), as well as all videos (https://zenodo.org/record/3608658). The machine learning approach is described in the Supplementary Materials and methods.

### Computer specifications and prior experience of experimenters

We used a Dell XPS 8930 workstation (Intel Core i7-8700K, 16GB RAM(DDR4), 512GB SSD, 2TB HDD, Nvidia GTX 1080 GPU) to implement the DLC-based approach, and to train the machine learning classifiers. We investigated the labeling, training, and analysis times of networks that use different numbers of labeled points. It takes an experienced experimenter ~5 min to label 20 frames with 18 points of interest (13 labels on the mouse and 4 or more labels on the arena, based on its complexity). Using the same computer described above, the network then trains overnight (ca. 11 h), and a 10-min video (928 × 576 pixels, 25 fps) is analyzed in ca. 9 min (see Supplementary Table S1). However, analysis/processing speed depends heavily on the hardware used, with GPU type and pixel number/frame size being of great importance [36].

### Behavior analysis

All annotators were trained by an expert behaviorist and reached a consensus on what constitutes each behavior before scoring any behavior. In the case of large discrepancies between annotators, the annotator in question was retrained, re-blinded and given the opportunity to score again. This was not the case for the live scoring, where the annotators initial values were reported. For detailed descriptions of behavior definitions see Supplementary Materials and methods.

### Statistical analysis

Data were tested for normality and all comparisons between normally distributed datasets containing two independent groups were performed using unpaired *t*-tests (two-tailed), whereas all comparisons between more than two groups were performed using one-way ANOVAs in order to identify group effects. Significant main effects were then followed up with post-hoc tests (Tukey’s multiple comparison test). We also report the coefficient of variation (CV) in order to show the dispersion of the data around the mean.

## Results

### Accurate animal tracking

Our goal was to compare the tracking performance of DLC to commercial solutions using three of the most popular rodent behavior tests in basic neuroscience research: the open field test, the elevated plus maze, and the FST. Robust tracking was previously demonstrated using DLC [17] and other open source tracking software (e.g. ezTrack) [37], thus we established DLC tracking in arenas that are compatible with commercial systems we routinely use in our lab. We labeled 13 standardized body points when tracking the mouse in each test (Fig. 1a). The labels relating to the arenas are particularly important (Fig. 1b–d), as they enable the calculation of standard parameters such as time spent in certain areas and distance traveled.

#### Open field

**Fig. 2:**
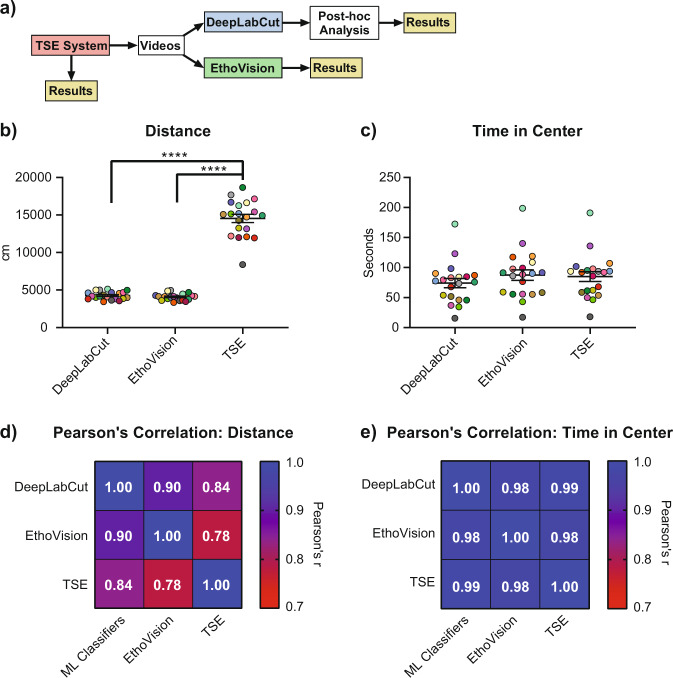
A comparison of basic tracking parameters in the open field test. **a** Schematic showing the workflow of the comparison between systems. **b**, **c** Distance and time in center as reported by DeepLabCut (with post-hoc analysis), EthoVision XT14, and the TSE Multi Conditioning System (TSE). **d**, **e** Correlation analysis of the performance of the different systems. Data expressed as mean ± standard error of the mean. Colors represent individual animals and are consistent across analysis techniques for direct comparison (*n* = 20) ****p < 0.0001.

We benchmarked DLC-tracking performance against commercial behavioral tracking solutions. Where possible, we scored each test using the “tracking-only” software EthoVision XT14 (Noldus), and the “all-in-one” TSE Multi Conditioning system. We tested 20 mice in the TSE Multi Conditioning System’s OFT arena, the videos acquired from these tests were then analyzed using EthoVision XT14 and DLC. In the OFT, simple tracking parameters such as distance traveled and time spent in zone (center) were comparable between DLC and EthoVision. However, TSE’s Multi Conditioning system reported a significantly different mean distance traveled (One-way ANOVA, *F*(2,57) = 331.9, *P* < 0.0001, CV = DLC:12.24%, EthoVision: 11.03%, TSE: 16.83%). TSE reported a similar value to that of DLC and EthoVision for time in center (CV time in center = DLC:46.28%, EthoVision: 45.05%, TSE: 43.09%) (Fig. 2). Heatmaps can also be plotted from all systems showing that time in zone is for the most part comparable (Fig. S2). The vastly different distance reported by the TSE system is likely due to its reliance on an infrared beam grid, which predicts the centerpoint of the animal based on the number and location of the beams that are broken. Thus, slight movement of the animal can lead to relatively large movements of the centerpoint, which could inflate the total distance traveled. This issue does not appear to affect the time spent in zones, since the fluctuation of centerpoint is unlikely to be large enough to move across zones. The distance recorded by the TSE system also correlates poorly with the other systems, thus we were concerned that such an inaccurate measure would lead to imprecise experimental results. To address this, we used a large cohort of mice (*n* = 59) available to us from another study, which were either normally reared in our facility (same 5 mice per cage after weaning, *n* = 29), or they were reared with high social exchange (new cage mates twice a week starting at weaning, *n* = 30). We tested these mice in adulthood in the open field test. When tracking with the DLC-based approach, we found that the social exchange group had higher locomotor activity (Fig. S3a, *t*(57) = 4.34, *q* = 0.004, multiple testing adjustment using Benjamini and Yekutieli correction) and spent more time in the center of the open field (*t* = 3.03, *q* = 0.015, Fig. S3b). When analyzed with the TSE system, ‘distance traveled’ did not reach statistical significance between groups (*t* = 2.57, *q* = 0.053, Fig. S3a), while time in center was significant (*t* = 3.07, *q* = 0.028, Fig. S3b). Therefore, the inaccurate distance tracking of TSE indeed occludes a clear biological effect. A power analysis shows that it would require 60 animals per group to achieve a 95% chance of successfully replicating the effect using the TSE system, but only 20 animals per group to replicate the effect with the DLC-based approach (Fig. S3e).

#### FST and EPM test

**Fig. 3:**
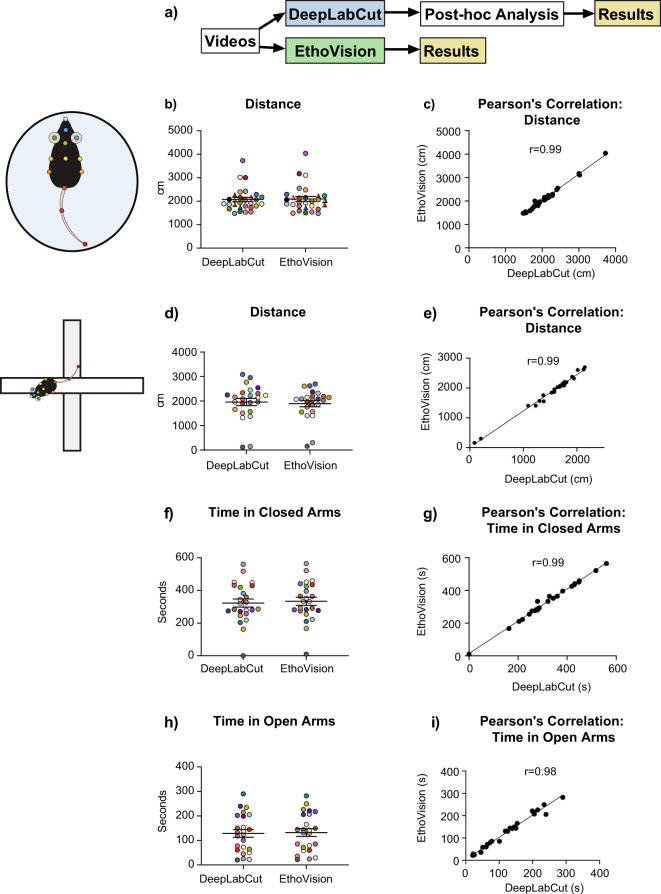
A comparison of basic tracking parameters in the forced swim test and elevated plus maze. **a** Schematic showing the workflow of the comparison between systems. **b**, **d**, **f**, **h** Basic tracking parameters in the forced swim test and elevated plus maze as reported by both DeepLabCut (with post-hoc analysis) and EthoVision XT14. **c**, **e**, **g**, **i** Correlation between the scores of the two systems. Data expressed as mean ± standard error of the mean. Colors represent individual animals and are consistent across analysis techniques for comparison (FST *n* = 29, EPM *n* = 24) *p < 0.05.

The FST and EPM analyses could not be scored using the TSE Multi Conditioning System, since the EPM/FST apparatus is not compatible with its “all-in-one” setup. We therefore acquired videos of 29 mice performing the FST, and 24 mice performing the EPM using EthoVision, which were later analyzed using DLC. Using DLC and EthoVision XT14, we found no significant differences regarding distance traveled in the FST or EPM (CV distance swim = DLC:23.71%, EthoVision:26.76%, CV distance EPM = DLC:32.75, EthoVision:32.74) or time in zones (CV Time in Open EPM = DLC:59.88, EthoVision:58.79; CV Time in Closed EPM = DLC:37.66, EthoVision:36.26; CV Time in Center EPM = DLC:55, EthoVision:56.73) (Fig. 3), again showing that both approaches can accurately track movement.

### Quantifying ethological behaviors with post-hoc analyses

#### Floating in the FST

**Fig. 4:**
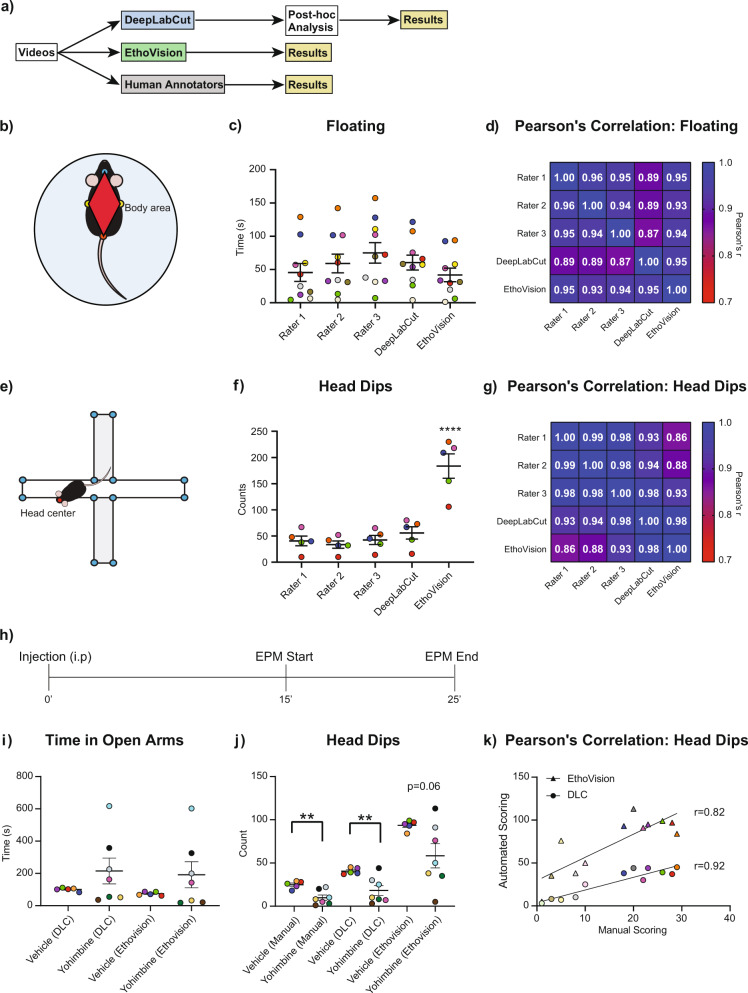
A comparison of quantifying ethological behaviors in the forced swim test and elevated plus maze. **a** Schematic of the workflow for the comparison between systems. **b**, **c** The polygon used in the definition of floating, and the body points taken into account when defining head dips. **d**, **e** Floating in the forced swim test and head dips in the elevated plus maze as reported by three human annotators (rater 1–3), DeepLabCut (with post-hoc analysis), and EthoVision XT14’s behavioral recognition module. **f**, **g** Correlation analysis for comparison between approaches. **h** Schematic showing the experimental design for yohimbine injections. **i** Time spent in the open arms after injection with yohimbine (3 mg/kg) or vehicle, as reported by DeepLabCut and EthoVision. **j** Head dips as reported manually, by DeepLabCut (with post-hoc analysis) and EthoVision. **k** Correlation analysis for comparison between approaches regarding head dips. Data expressed as mean ± standard error of the mean. Colors represent individual animals and are consistent across analysis techniques for comparison (FST *n* = 10, EPM *n* = 5) **p < 0.01, ****p < 0.0001.

Providing evidence that DLC can perform basic tracking functions similarly to commercial software/hardware packages, we next attempted to score ethological behaviors using the coordinates for each datapoint tracked by DLC. Animals were considered to be floating depending on the rate of change of the polygon “body area” (Fig. 4b). To establish the best possible ‘ground truth’, three human annotators manually scored floating behavior in a set of ten FST videos. Using the same videos, we were able to accurately identify floating behavior (Fig. 4d). In addition, we compare this to the ‘activity’ module for EthoVision XT14, which can be used to score floating behavior. We detected no significant differences in time floating, with EthoVision showing a better correlation with manual scoring than DLC.

#### Head dips in the elevated plus maze

In the EPM, we recorded head dips—an exploratory behavior—using DLC and EthoVision (Fig. 4d). Here we saw significant group effects (One-way ANOVA, *F*(4,20) = 23.82, *P* < 0.0001, CV head dips = R1:50.71 %, R2:46.38 %, R3:45.54 %, DLC:47.03 %, EthoVision:28.33%), with differences between all groups and EthoVision (Tukey’s multiple comparisons test, *q* = 10.96(R1), 11.50(R2), 10.83(R3), 9.60(DLC), df = 20, *p* < 0.0001), but no differences between human annotation and DLC. To test whether these differences have direct biological relevance, we injected a small cohort of mice with either 3 mg/kg yohimbine, an alpha2-adrenoceptor antagonist known to trigger anxiety through increased noradrenaline release in the brain [38, 39]. Fifteen minutes after injection, mice were tested on the EPM (Fig. 4h). We observed no significant differences between the time both groups spent on the open arm, with either our DLC approach or EthoVision (Fig. 4i). However, when measuring head dips, both manual scoring and our DLC-based analysis detected a significant reduction in head dips after yohimbine injection (manual: *t* = 3.68, df = 10, *p* = 0.004; DLC: *t* = 3.21, df = 10, *p* = 0.009), showing the expected decrease in exploratory behavior associated with increased anxiety. In contrast, EthoVision failed to detect a significant group effect (*t* = 2.08, df = 10, *p* = 0.064.) (Fig. 4j). This is likely due to EthoVision’s lower correlation to manual scoring (*r* = 0.82 as opposed to 0.92 with DLC-based tracking) (Fig. 4k), presumably caused by an inappropriate parametric definition of head dip behavior, which is addressed in more detail in the discussion.

#### Flexible tracking in a variety of scenarios

To further demonstrate the versatility of our approach, we extended our tracking analysis to two tests that rely on accurate tracking of the animal in slightly more complex environments, the 3 Chamber Sociability Test (3CST) and the Barnes Maze. Neither of these tests are available for static systems like the TSE setup, but can be scored with EthoVision. The 3CST assesses sociability in mice, by measuring how much social approach/interaction a freely moving mouse displays toward an enclosed conspecific [40]. We demonstrate that the amount of social interaction (defined as time the tip of the nose is in the interaction zone) is highly correlated between the DLC-based tracking and EthoVision tracking (Pearson’s *r* = 0.95, *p* < 0.001, Fig. S4c), and that mice spend more time investigating the enclosure containing the conspecific than the enclosure with a novel object (Fig. S4). Similarly, the Barnes Maze relies on accurate tracking to test how fast an animal acquires a spatial memory by finding an escape tunnel or a food reward in one of many holes placed around a circular platform [41]. Our approach precisely tracks the movement of a mouse across the maze, and records the time it spends investigating different holes (Fig. S5).

We then leveraged the increased flexibility and accuracy of our approach to quantify changes in head angle, using tracking data from nose, neck, and tailbase (see Fig. S6b), which is currently impossible to track with commercially available systems. Head angle is an important, biologically relevant measure in many different circumstances (e.g., object exploration tasks or swim direction in the Morris water maze). Here, we aimed to quantify the rapid changes in head angle induced by optogenetic stimulation of striatal D2 medium spiny neurons. This manipulation is well-known to elicit ipsiversive (clockwise) head rotations, which can lead to full-body rotations when the animal is moving. Full-body rotations can be quantified with commercial systems [42–44], yet it is not possible to continuously track the mouse head angle over time, which hampers the quantification of subtle changes driven by D1/D2 excitation:inhibition imbalances. Our analysis shows that head-turns (up to 40°) are immediately triggered by optogenetic stimulation, and quickly disappear after stimulation has terminated (Fig. S6c). This effect is robust and it can be reliably quantified at the single-subject level (Fig. S6d), as well as at the group level (Fig. S6e)

### Quantifying ethological behaviors with machine learning

#### Floating in the FST

So far, we have demonstrated that manually defined parameters can be used to automatically determine distinctive behaviors based on custom-defined criteria and simple post-hoc scripts to analyze tracking data generated by DLC. However, we found that using this approach for more complex behaviors was labor intensive, arbitrary and sometimes inaccurate, as exemplified by the fact that this ad-hoc approach could not outperform the floating analysis performed by EthoVision (see Fig. 4d). Therefore, we first determined whether we could improve the detection of floating behavior by training a supervised classifier on the acceleration of all body points. To gain enough data to train a neural network, we increased the number of labeled videos provided by one experimenter to 20. We then trained a small artificial neural network (2 layers, L1 = 256 neurons, L2 = 128 neurons, fully connected) to recognize short sequences of body point accelerations during epochs of floating and not floating. We used the data of one annotator (Rater 1) to train ten behavior classifiers. To cross validate classification performance we trained each classifier on 19 videos and then tested on the remaining video. We cross validated with the same videos that were used for Fig. 4d. The new classifier showed the highest correlation with human labeling of Rater 1 (Pearson’s *r* = 0.97), thus outperforming the manual cutoff (Pearson’s *r* = 0.89) and EthoVision (Pearson’s *r* = 0.95). The classifier also outperformed EthoVision when compared to the other two raters (Fig. S7b). Notably, the classifier showed a similar correlation to other human raters as the original rater, indicating that it performed just as well (Fig. S7b). Interestingly, when taking the average of all raters (“Rater Average”, Fig. S7b), the correlation of both EthoVision and the new classifier each reach near perfect correlation (*r* = 0.99), strongly suggesting that individual human raters are less reliable than well-calibrated automated approaches.

#### Rearing in the open field test

We then applied supervised machine learning to recognize complex behaviors in the open field test. We used the coordinates for each datapoint tracked by DLC to reconstruct a rotation and location invariant skeletal representation of the animal (Fig. S8). We then trained a small artificial neural network (as described above) to recognize short sequences of the skeletal representation during epochs of supported and unsupported rears. We focused on rearing in the open field since supported and unsupported rears are very similar movements (both include standing on hind legs), which are difficult to score automatically [6]. Again, we had three annotators scoring 20 videos (10 min long) to set the ground truth for rearing frequency, and annotate the exact onset and offset of each behavior. We used the data of each annotator to train 20 behavior classifiers. To cross validate classification performance we trained each classifier on 19 videos and then tested on the remaining video. This allowed us to assess the classifier’s performance and to calculate correlation to the human annotation. Overall our behavior classifiers reached a frame-to-frame accuracy of 86 ± 3% (Fig. S9). No significant differences were observed between any of the human investigators (R1–3) or the machine learning classifiers trained using their data (MLR1–3) in the scoring of either supported rears (CV = 16.41% (R1), 16.04% (R2), 19.04% (R3), 15.75% (MLR1), 16.85% (MLR2), 17.23% (MLR3)) or unsupported rears (CV = 50.33% (R1), 48.86% (R2), 45.84% (R3), 47.76% (MLR1), 50.67% (MLR2), 42.43% (MLR3)). Therefore, supported and unsupported rearing can be measured as accurately by supervised machine learning algorithms as by human manual scoring, the gold standard in the field (Fig. S10). To address whether our approach can be directly implemented in female mice and in animals of different ages, we randomly selected 4 OFT tests that had previously been conducted in females under the same conditions in our lab. We found a high correlation and significance between the manual and automated scoring of supported rears (*r* = 0.968, *p* = 0.032) and unsupported rears (*r* = 0.969, *p* = 0.032) also in females (Fig. S11). Notably, two of the female mice were 3 months old at the time of testing, and two were ~6 months old, indicating that the algorithm appears to be reliable also when testing mice of different ages and sizes.

**Fig. 5:**
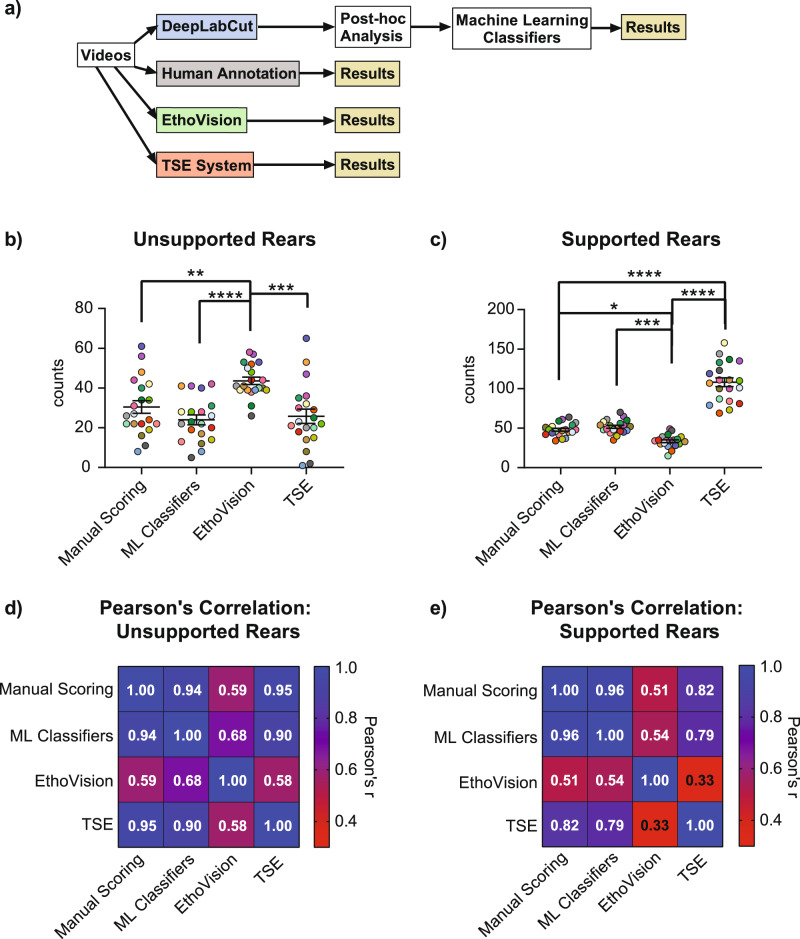
A comparison of complex behavioral scoring between human raters, machine learning classifiers and commercially available solutions. **a** Schematic of the workflow. **b**, **c** Unsupported and supported rears in the open field test as reported by three human raters (averaged and plotted as manual scoring) and three machine learning classifiers (averaged and plotted as ML classifiers), EthoVision XT14 and the TSE Multi Conditioning System (TSE). **d**, **e** Correlation analysis for comparison. Data expressed as mean ± standard error of the mean. Colors represent individual animals and are consistent across analysis techniques for comparison (*n* = 20). *p < 0.05, **p > 0.01, ***p < 0.001, ****p > 0.0001.

We then returned to the male data to compare performance between our approach and the commercial systems. We took the mean score from the human investigators and the mean score from the machine learning classifiers for each type of rearing and compared them to those reported by the TSE Multi Conditioning System, which includes a separate infrared tracking grid (z-grid, which counts beam-breaks as rears) and to EthoVision’s behavior recognition module (Figs. 5 and S10). Significant group effects were observed in the scoring of unsupported rears (one-way ANOVA, *F*(3,76) = 9.547, *p* < 0.0001) with differences between the human raters and EthoVision (Tukey’s multiple comparison test, *q* = 4.590, DF = 76, *p* = 0.0093), the machine learning-based behavioral classifiers and EthoVision (Tukey’s multiple comparison test, *q* = 6.841, DF = 76, *p* < 0.0001), and between EthoVision and TSE (Tukey’s multiple comparison test, *q* = 6.213, DF = 76, *p* = 0.0002). We observed significant group differences between the number of supported rears reported by EthoVision, TSE, and the human and machine learning classifiers (one-way ANOVA, F(3,76) = 104.5, *p* < 0.0001). Post-hoc tests reveal significant differences between the human raters and EthoVision (Tukey’s multiple comparison test, *q* = 4.518, DF = 76, *p* = 0.0108), and between the human annotators and the TSE system (Tukey’s multiple comparison test, *q* = 18.72, DF = 76, *p* < 0.0001). Similarly, the machine learning classifiers reported significantly different results to those reported by EthoVision (Tukey’s multiple comparison test, *q* = 5.670, DF = 76, *p* = 0.0008) and the TSE system (Tukey’s multiple comparison test, *q* = 17.57, DF = 76, *p* < 0.0001). The TSE system and EthoVision were also in disagreement (Tukey’s multiple comparison test, *q* = 23.24, DF = 76, *p* < 0.0001). Again, no significant difference was detected between the performance of the humans or machine learning classifiers, which were highly correlated (Fig. 5d, e). We conclude that EthoVision reports an inaccurate number of unsupported rears, while both EthoVision and TSE perform very poorly on supported rears. It is important to note that we spent a considerable amount of time and effort calibrating the TSE system specifically to report unsupported rears accurately. However, it appears that the TSE system cannot score both supported and unsupported rears accurately at the same time. In contrast, the supervised machine learning-based behavior classifiers performed as well as the human annotators, the gold standard in the field.

## Discussion

This report shows that DeepLabCut (DLC) video tracking combined with simple post analyses can detect and quantify behavioral data as well as—if not better than—commercial solutions. Moreover, we developed supervised machine learning approaches that use features extracted from the DLC-tracking data to score complex, ethologically relevant behaviors. We demonstrate that the machine learning classifiers approach human accuracy, while outperforming commercial systems at a fraction of the cost. We show that the flexibility and accuracy of our approach can increase statistical power by reducing variability, and that it can be readily applied to a wide variety of different setups and more specialized measurements.

Scoring complex ethological behaviors with human-like accuracy is an important step forward in the analysis of behavior [4, 15, 45–48]. Commercial attempts to automatically score ethological behaviors have reduced intra-rater variability and increased throughput, but at the cost of accuracy. The machine learning approaches used here are capable of reducing intra-rater variability by eliminating factors such as fatigue or human bias, whilst scoring with similar accuracy as trained human investigators. Analyzing “head dips” or “rearing” requires the purchase of an additional module from EthoVision. In addition, commercial packages often give explanations as to how they define their parameters, but these definitions are not consistent between different commercial solutions and cannot be altered. These differences are likely the reason that EthoVision scores head dips so poorly in comparison to human investigators (Fig. 4). EthoVision also poorly scored grooming behavior [4], although the reason for the poor performance often remains unclear, since the code is not open source. Similarly, the TSE system, which relies on infrared beam grids, is less flexible, it cannot distinguish between different behaviors that may break the z-grid beam and is therefore inaccurate. It is important to highlight that commercial systems allow altering the analysis parameters (to varying degrees). For the purposes of this report we tried to use the default/suggested settings where possible, and invested approximately equal amounts of time into the setup of all systems, thus giving a representable comparison whilst acknowledging that the performance of any system could still be improved (within limits). The data presented here show that inaccurate tracking provided by grid-based, commercial tracking systems (TSE) can occlude highly significant behavioral differences between experimental groups (Fig. S3), and that it would require a cohort of mice three-times larger to reveal the group difference using the TSE system. Video analysis packages such as EthoVision also have limitations, for instance EthoVision requires the test arena to be defined prior to analysis. Once the test arena has been defined this is no longer flexible, meaning that if the apparatus is moved slightly during cleaning it has to be returned to exactly where it was when the arena was defined. Although seemingly only a minor issue, this can drastically increase the amount of time required to score videos in which the camera/arena moved slightly, which can easily happen during cleaning. Since DLC-tracking also detects the arena, it is impervious to these slight movements and the calibration of the arena is always optimal regardless of the size of objects in the video, making it less prone to errors when the setup is used for multiple tests. DLC could also prove useful when working under more ethological conditions in arenas with bedding material/variable backgrounds. In these settings commercial solutions will likely struggle even more, while the powerful deep-learning approaches will get to flex their muscles.

Beyond the behaviors investigated here, researchers are of course interested in many different behaviors such as stretching, grooming, or social interactions. While TSE cannot detect any of these behaviors, EthoVision can detect them with some degree of accuracy [49], after the individual behavioral recognition modules have been purchased. However, approaches based on point-tracking data can be used to identify any behavior of interest either by defining simple post-analysis parameters (e.g., elongation of body-length vectors to detect streching), or by training machine learning classifiers. This not only saves time but also money as similar approaches can be used to score any number of behaviors at no cost, and ensure consistency within and between labs. While machine learning has been used successfully to score complex ethological behaviors before [for review, see [2]], the major innovation of our approach is that the input features are based on tracking using DLC [17, 18]. DLC offers extremely precise tracking of individual body parts, and is amongst the most widespread and most user-friendly deep-learning-based pose estimation tools [50–53]. In our opinion, the exact choice of pose estimation software is not essential, although two recent preprints have similarly opted to use DLC data for automated recognition of complex rodent behaviors [54, 55].

Another key advantage of using pose estimation software to generate the input data for machine learning classifiers is that it offers increased tracking flexibility by enabling users to define and record the parameters of interest themselves. In contrast, commercial systems have unnecessary constraints or paywalls in place. Although our training set did not contain sufficient instances of grooming behavior to train a classifier (only 20–30 events in our entire training dataset), a recent report used transgenic mice that show an over-grooming phenotype to train machine learning algorithms to accurately quantify grooming behavior, vastly outperforming EthoVision [4]. Recently, DLC-based tracking was combined with unsupervised clustering to reveal different subtypes of grooming (face groom, head groom etc.) [54]. In addition, complex social behaviors can be analyzed by combining point-tracking with supervised machine learning [55]. This confirms the enormous potential of approaches like ours, which couple pose estimation data with machine learning classifiers [2]. Notably, we have chosen a dense skeletal labeling approach (13 body points) in order to have maximal flexibility when scoring complex behaviors in multiple setups, yet similar approaches have achieved very good accuracy with only 8 or fewer labeled body points [54, 55].

Regarding human scoring, our annotators were all trained at the same time by an expert behaviorist, and reached a consensus about what constituted each behavior before beginning to score the videos. In the case of notable discrepancies between annotators, the annotator in question was trained again, re-blinded and given the opportunity to re-score the videos again. This reduced inter-rater variability that can arise from differences in the definitions of the behaviors even within a given lab, or from subtle differences in human judgment [4, 10, 11]. In addition, the behaviors reported here were not scored in live, but offline, which enabled stop-and-play analysis of videos for frame-by-frame labeling. This offers advantages over live scoring, especially regarding fast or complex behaviors. We show that human accuracy drops when scoring behavior videos in real time (live), and that behaviors that occur in quick succession are particularly challenging for human raters (see Fig. S10 for live vs. offline behavioral scoring comparisons). Together, these factors likely explain why our inter-annotator scoring correlations are higher than some of those previously reported (approximately *r* = 0.96 instead of *r* = 0.90 for floating [9, 56]). Although offline labeling with this level of accuracy is extremely time consuming (~1 h per 10-min video), once the machine learning classifiers have been trained, no further manual scoring is required, thus reducing the overall time and effort required to accurately score behavior in the future.

As behavioral analysis moves more toward video tracking as opposed to reliance on beam grids, recent developments in unsupervised behavioral identification approaches have widened the horizons of what was previously thought possible [1, 2]. Approaches that focus on the unsupervised identification and separation of behavioral patterns are beginning to reveal the true complexity and richness of animal behavior [12, 13, 16, 54], However the interpretation of the findings from unsupervised machine learning techniques are more difficult. Although impressive, the implementation and use of many of these unsupervised behavior recognition approaches is out of reach of many basic science labs that lack the necessary programming and machine learning know-how. Therefore, widespread use/dissemination of new cutting-edge techniques will likely depend on their commercialization as part of user-friendly software/hardware solutions. In contrast, modern deep learning/machine vision-based tracking and behavioral identification approaches such as those demonstrated here using DeepLabCut, are already taking over the field of behavioral neuroscience. Efforts are currently underway to generate user-friendly free software tools to facilitate the implementation of markerless point-tracking with machine learning approaches [54, 55]. Such advances are poised to revolutionize the ease and consistency with which rodent behavior can be quantified in labs across the world. In this first systematic, head-to-head comparison, we show that such approaches outperform commercial systems, achieve human-like accuracy and surpass human reliability, all while being fully automated, flexible, and affordable.

## Funding and disclosure

This project was funded by the ETH Zurich (JB and BG), the ETH Project Grant ETH-20 19-1 (JB and BG), the SNSF Grants 310030_172889/1 (JB) and CRSII5-173721 (BG), the Forschungskredit of the University of Zurich FK-15-035 (JB), the Swiss Data Science Center C17-18, (BG), the Vontobel-Foundation (JB), the Novartis Foundation for Medical Biological Research (JB), the EMDO-Foundation (JB), the Olga Mayenfisch Foundation (JB) and the Betty and David Koetser Foundation for Brain Research (JB), and two Neuroscience Center Zurich Project Grants Oxford/McGill/Zurich Partnership (JB and BG). Open access funding provided by ETH Zurich. The authors declare no conflict of interest.

## Supplementary information

Supplementary Data

Supplementary Material and Methods
