# Supplementary material for: Deep learning-based behavioral analysis reaches human accuracy and is capable of outperforming commercial solutions
Source: Neuropsychopharmacology. 2020 Jul 25;45(11):1942–52. doi: 10.1038/s41386-020-0776-y (PMC7608249; doi:10.1038/s41386-020-0776-y)
Supplement: Supplementary file 1 — Supplementary Data [file 41386_2020_776_MOESM1_ESM.docx]

**SUPPLEMENTARY DATA**

**Deep learning based behavioral analysis reaches human accuracy and is capable of outperforming commercial solutions**

**Oliver Sturman^1,2^, Lukas von Ziegler^1,2^, Christa Schläppi^1,2^, Furkan Akyol^1,2^, Mattia Privitera^1,2^, Daria Slominski^1,2^, Christina Grimm^2,3^, Laetitia Thieren^2,4^, Valerio Zerbi^2,3^, Benjamin Grewe^2,5,6^, Johannes Bohacek^1,2^**

^1^ Laboratory of Molecular and Behavioral Neuroscience, Institute for Neuroscience, Department of Health Sciences and Technology, ETH Zurich, Switzerland

^2^ Neuroscience Center Zurich, ETH Zurich and University of Zurich, Switzerland

^3^ Neural Control of Movement Lab, Department of Health Sciences and Technology, ETH Zürich, Switzerland

^4^ Experimental Imaging and Neuroenergetics, Institute of Pharmacology and Toxicology, University of Zurich, Switzerland

^5^ Institute of Neuroinformatics, University of Zurich and ETH Zurich, Switzerland

^6^ Department of Information Technology and Electrical Engineering, ETH Zurich, Switzerland

**Supplementary Tables and Figures**

| **Number of Points of Interest Tracked** | **Time taken to label 20 frames** | **Time taken to train network (250,000 iterations)** | **Time taken to analyze a 10 minute video** |
| --- | --- | --- | --- |
| **3** | **1:46** | **11:15:00** | **8:40** |
| **6** | **1:55** | **11:20:00** | **8:43** |
| **12** | **4:12** | **11:13:00** | **8:55** |
| **18** | **5:23** | **11:10:00** | **9:00** |

**Table S1. Comparison of the labelling, training and analysis speeds of networks with varying numbers of labeled body points.** We created four new open field test (OFT) networks, each trained to track a different number of points of interest. After the frames had been labelled and the networks had been trained, we used each network to analyze a test video (928 x 576 px, 25 fps) in order to compare the analysis speed.

**
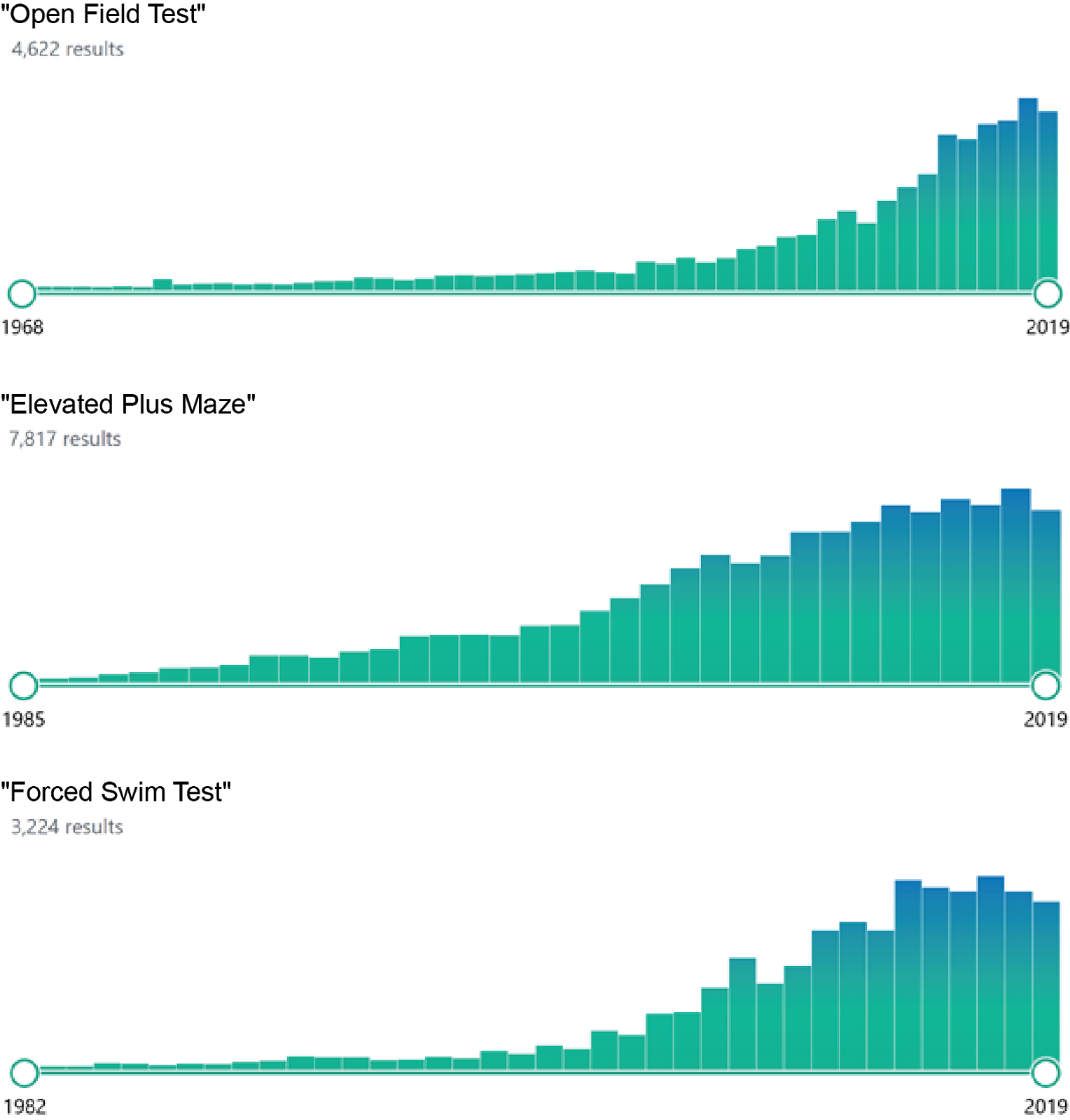
**

**Figure S1. The number of citations relating to the Open Field Test, Elevated Plus Maze and Forced Swim Test.**  Over the last few decades the popularity of these behavioral assays has increased dramatically, making them three of the most popular behavioral assays used in behavioral neuroscience.


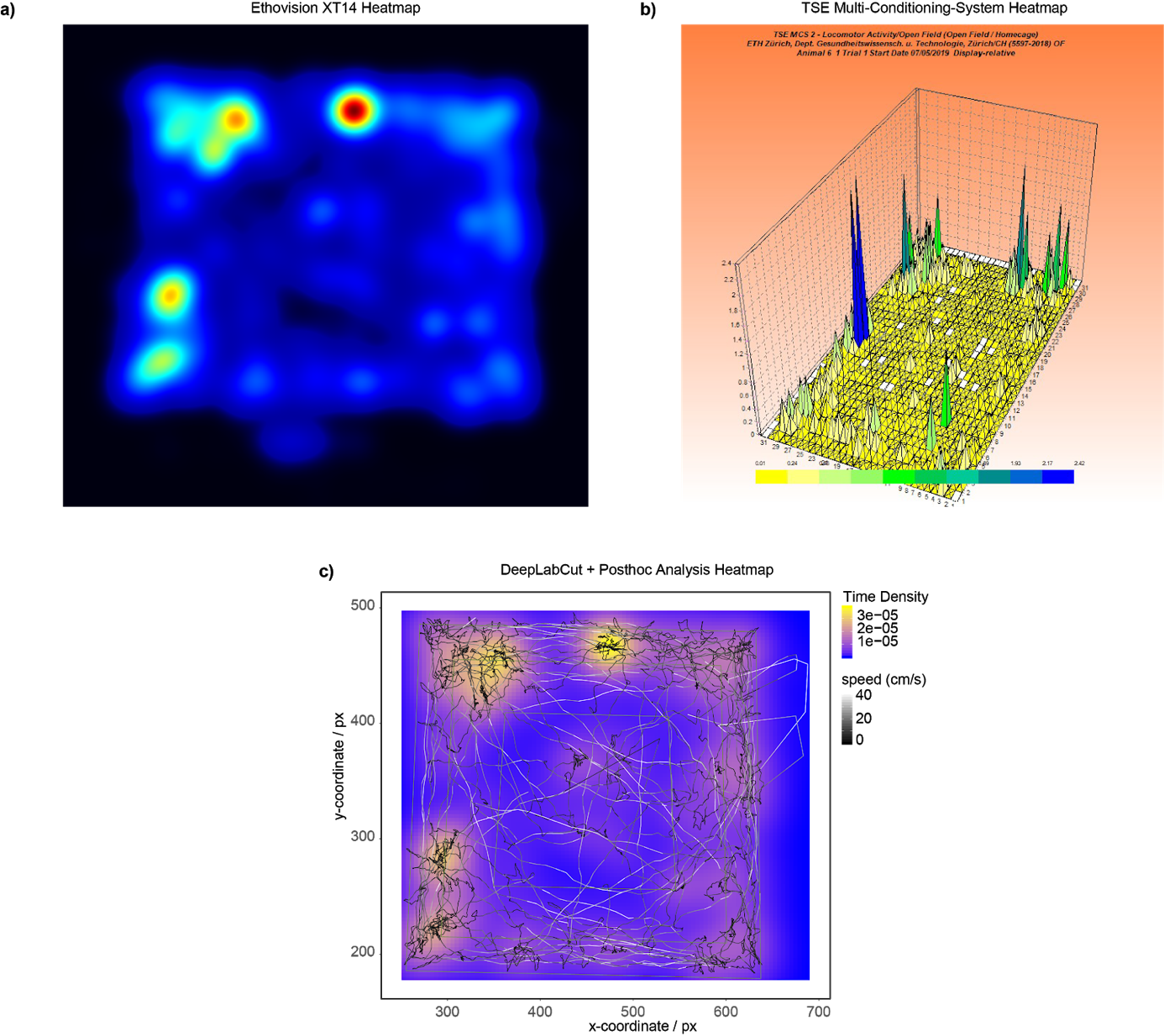


**Figure S2. Heatmaps.** example heatmaps generated using **(a)** EthoVision XT14, **(b)** TSE’s Multi Conditioning System and **(c)** DeepLabCut (with post hoc analysis and ggplot2).

**
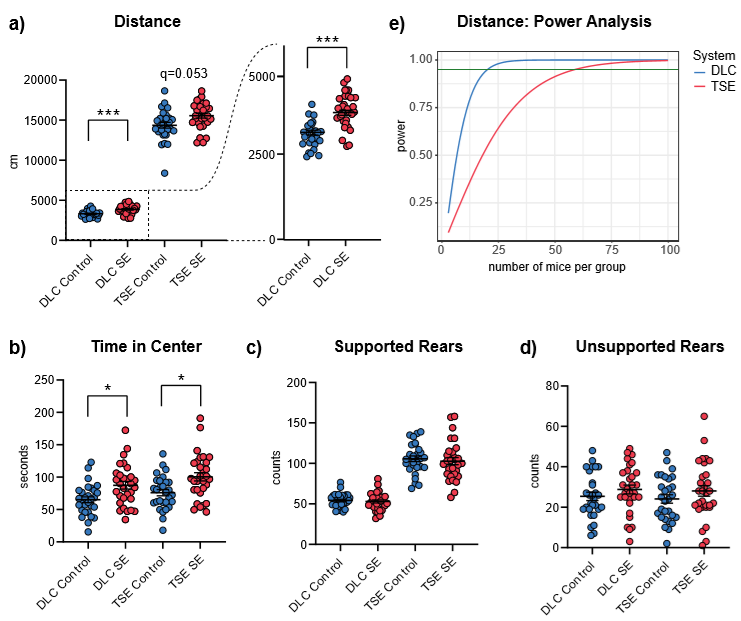
**

**Figure S3. Deep learning approaches reveal biological effects and increase statistical power in comparison to a commercial tracking system.** Two groups of mice were either reared under standard conditions (Control; 5 mice per cage) or exposed to frequent social exchange (SE) for 7 weeks (5 new mice twice a week) starting at weaning (n=29-30 mice per group). **(a)** Direct comparison of the same video recordings shows that the DeepLabCut-based (DLC) tracking finds a highly significant increase in distance travelled for the social exchange group (t(57)=4.34, q=0.0004), whereas tracking with the commercial solution (TSE Systems Multi Conditioning Chamber, Germany) fails to identify a significant effect (t(57)=2.57, q=0.053). **(b)** A significant difference between time in center is detected by both the DLC-based approach (t(57)=3.03, q=0.015) and the TSE analysis (t(57)=3.07, q=0.028). **(c,d)** Both systems detect no group differences between the other recorded behavioral parameters, supported rears (DLC t(57)=0.47, q=>0.99, TSE t=0.51, q=>0.99) and unsupported rears (DLC t(57)=1.14, q=0.72, TSE t(57)=1.19, q=0.67).*=p<0.05, ***=p<0.001. **(e)** Power analysis reveals that using the DLC-based approach increases statistical power. To replicate the same effect with 95% confidence, three times fewer animals have to be used with the DLC-based approach (n=20) compared to the TSE system (n=60).

**
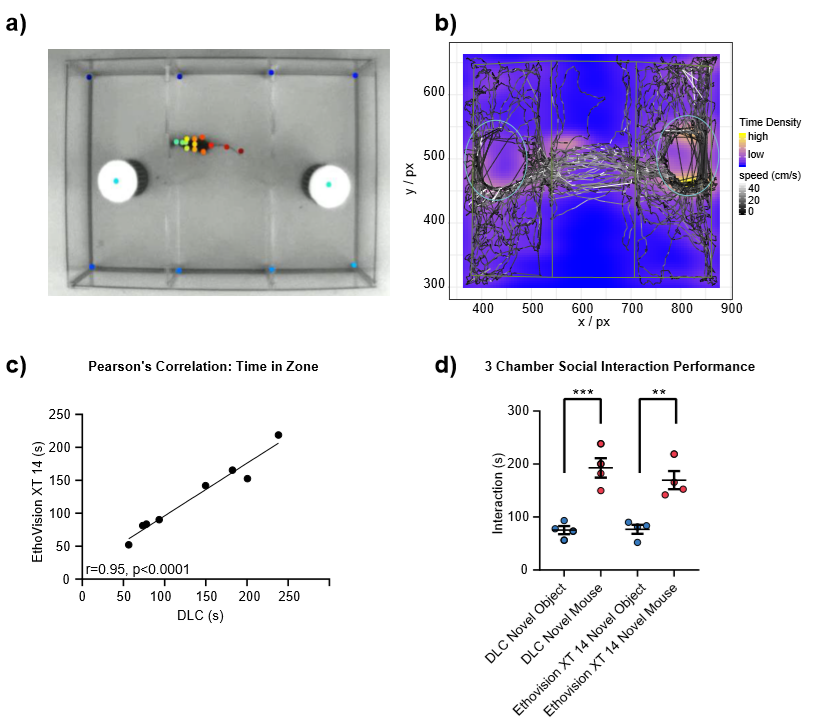
**

**S4. Proof of concept for the use of the DLC-based tracking approach in the Three-chamber Sociability Test (3CST).** Four mice of varying ages (9-26 weeks) were placed into the 3CST chamber and given the choice between investigating a novel object or novel mouse. **(a)** The standardized points of interest used to track the animal in the 3CST. **(b)** The heatmap produced by tracking the nose point of a single animal in one 10-minute trial, indicating both time in zone and speed. **(c)** The correlation between the time investigating both the novel object and novel mouse from both EthoVision XT 14 and the DLC-based approach. **(d)** Mice spend more time investigating the novel mouse than the novel object, an effect that is similarly detected by EthoVision XT 14 (t(12)=4.76, p=0.0028) and the DLC-based approach (t(12)=6.025, p=0.0004). **=p<0.01; ***=p<0.001

**
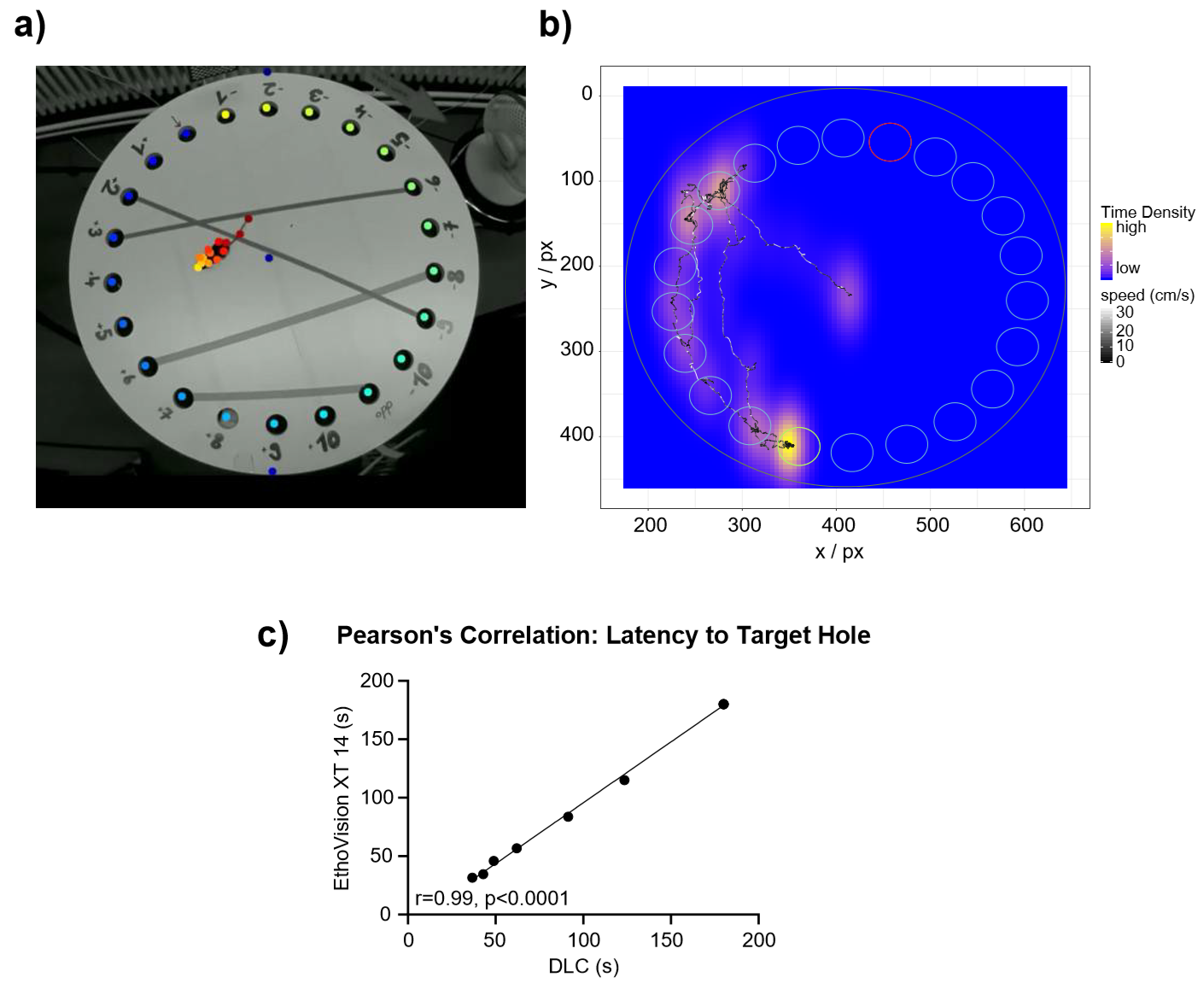
**

**S5. Proof of concept for the use of the DLC-based approach in the Barnes Maze.** Two mice were placed onto a Barnes maze and tasked with finding the target hole over several sessions. **(a)** The standardized points of interest used to track the animal in the Barnes Maze. **(b)** The heatmap produced by tracking the nose point of a single animal in a 3-minute trial, indicating both time in zone and speed (target=light green circle, opposite=red circle). **(c)** The correlation between the latency to reach the target hole from both EthoVision XT 14 and the DLC-based approach.


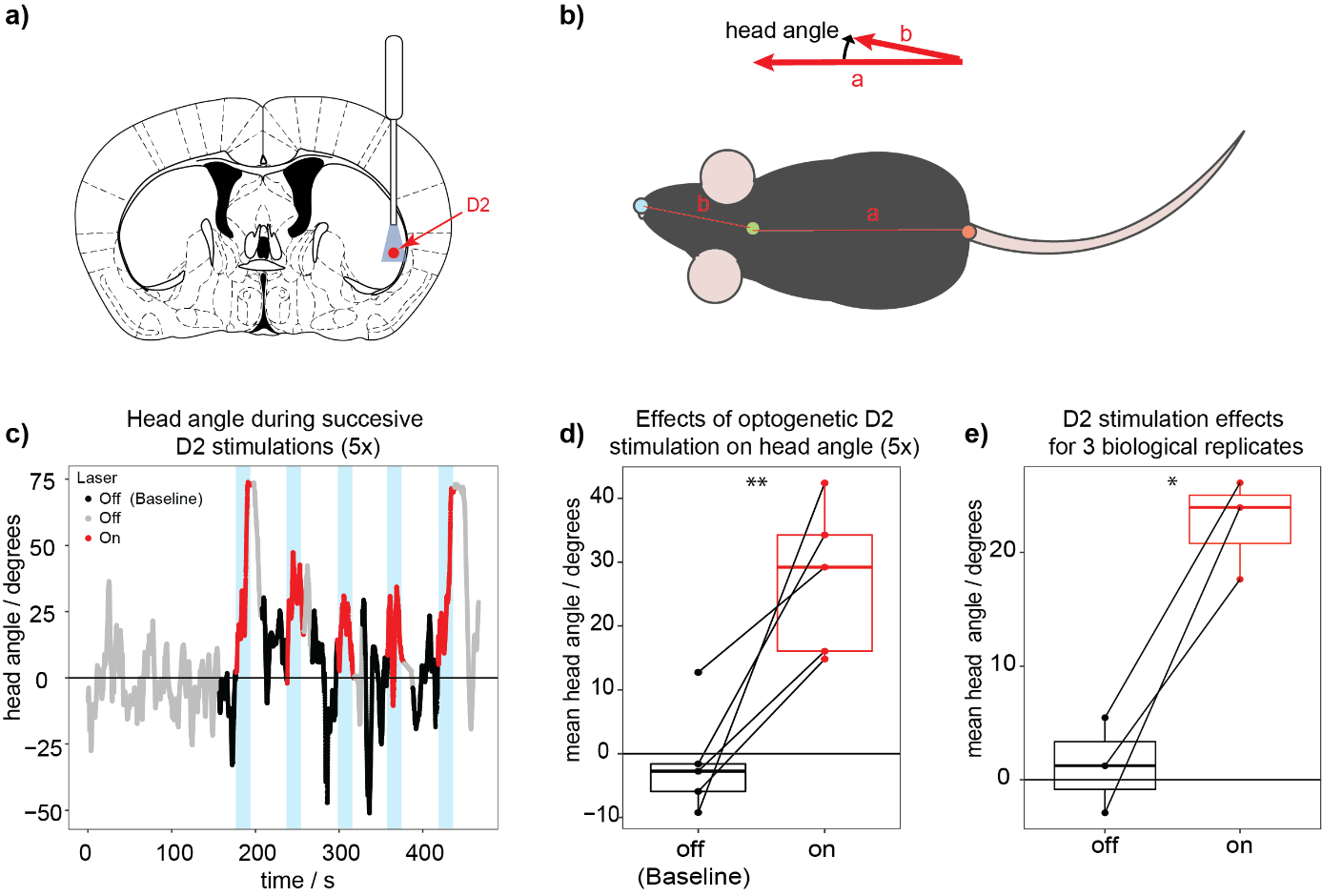
 **Figure S6. Tracking head angle during optogenetic stimulation of striatal D2 medium spiny neurons**. **(a)** Schematic showing the site of optogenetic stimulation. **(b)** The body points used to calculate the head angle. **(c)** Head angle over time during a single trial. Optogenetic stimulation is highlighted using blue bins and red trace. The period after stimulation is highlighted with a dark black trace. **(d)** Effects of 5x optogenetic stimulation of a single animal **(e)** The mean difference in head angle during laser on/off times of 3 mice.


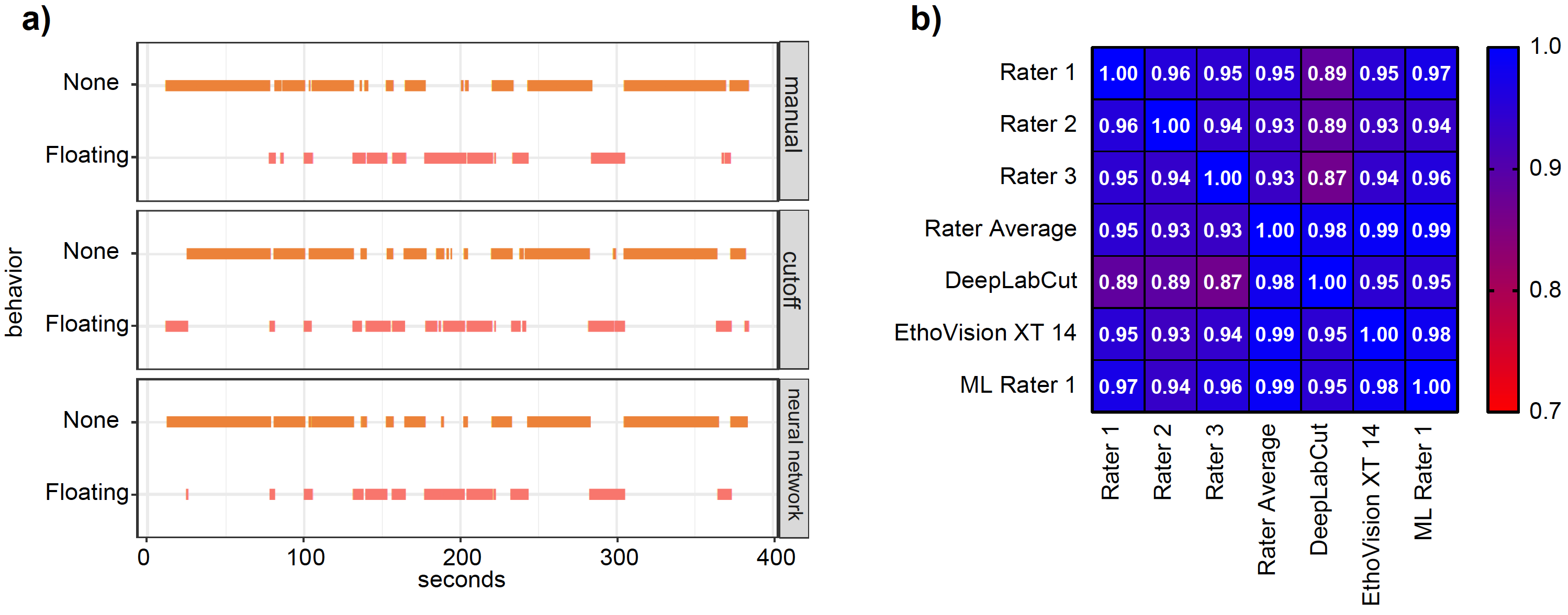


**Figure S7**. **Supervised machine learning analysis of floating behavior in the forced swim test. (a)** example of one single FST recording for which floating behavior was rated either manually (top panel), using an ad-hoc movement cutoff based on change of mouse body shape as described in Figure 4 (middle panel), or using a supervised machine learning approach (neural network) trained on other videos (bottom panel). This example shows very good congruency between all three different approaches, but also indicates instances where the different approaches diverge (e.g. in the first 50 seconds). **(b)** Correlation analysis (using Pearson’s r) between the different raters when applied to the total floating time from the same 10 videos that were used for Figure 4. The ML Rater 1 shows the performance of the neural network that was trained with data from rater 1. Each video was tested with a network that was trained on a training set that did not include said video.

**
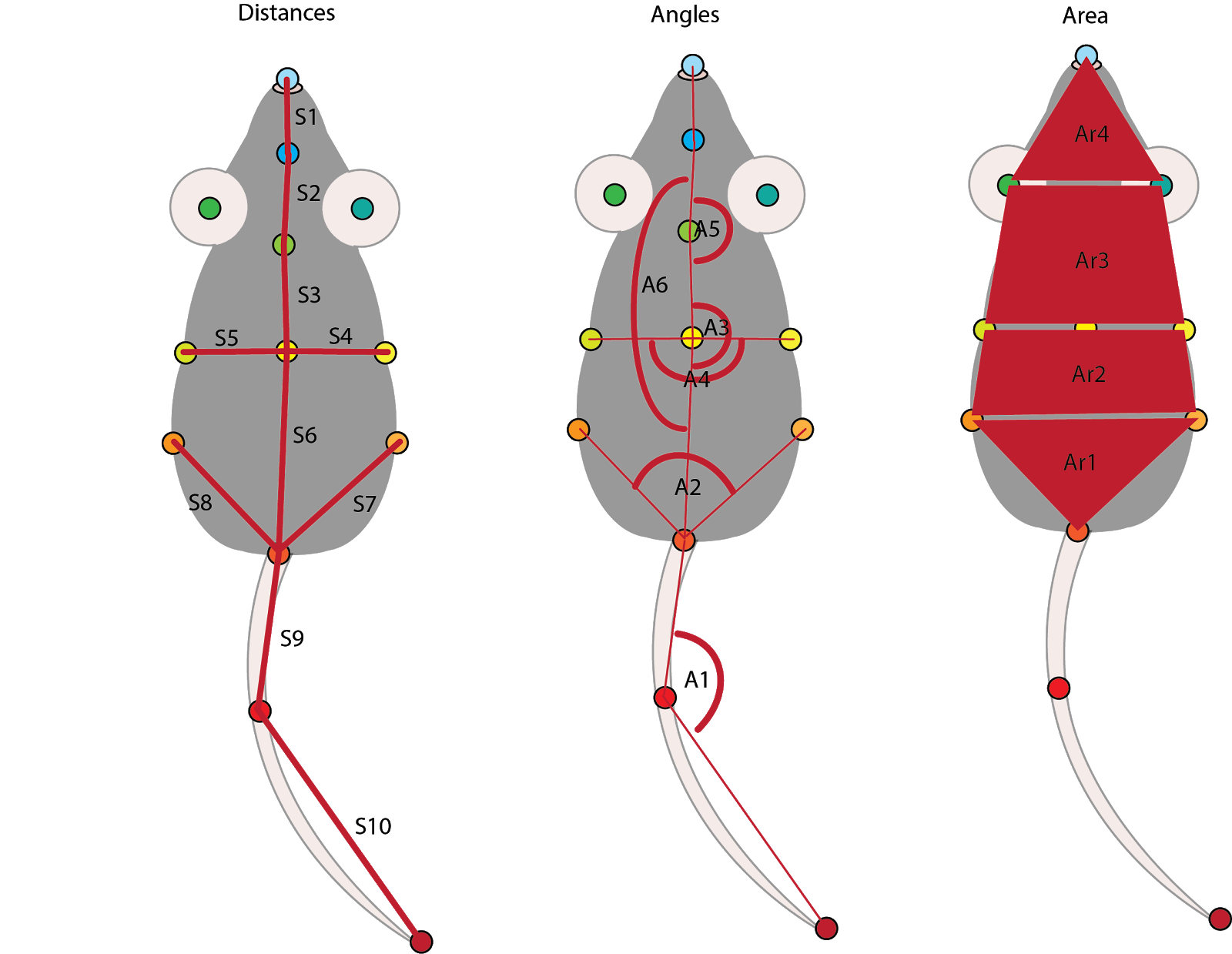
**

**Figure S8. Features used for machine learning.** The skeletal information taken into account during the machine learning process, gathered from the tracking data obtained using DeepLabCut.


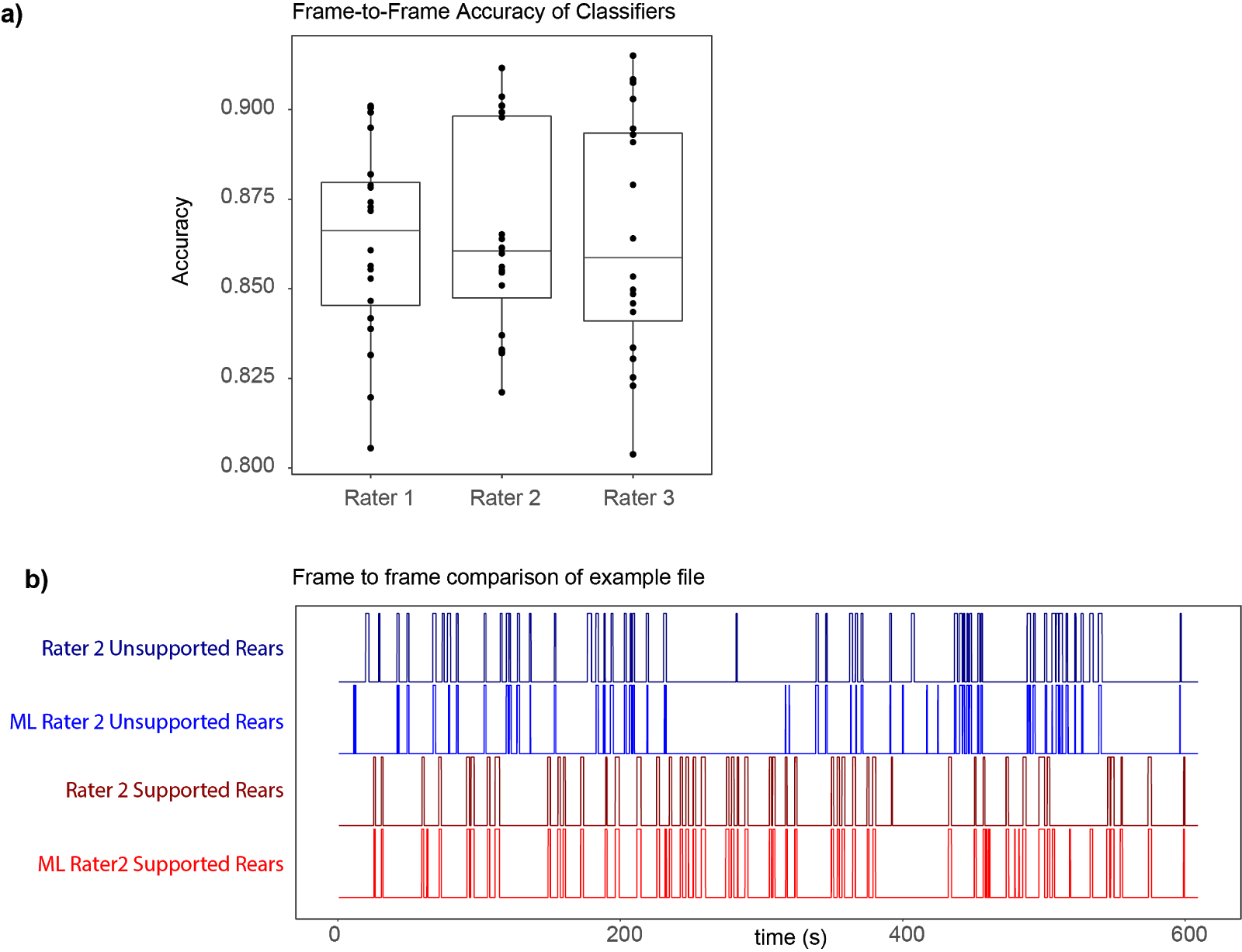


**Figure S9.** **Frame by frame comparison of scoring by a single human rater and their machine learning counterpart**. **(a)** The frame-by-frame accuracies of each rater and the behavioral classifier trained using their labelling data. **(b)** An example of the frame-by-frame labelling, each bar indicates the onset and offset of the indicated behavior. Although some inconsistencies can be observed, the total number of behaviors reported is similar with a frame-to-frame accuracy of 86 ± 3%.


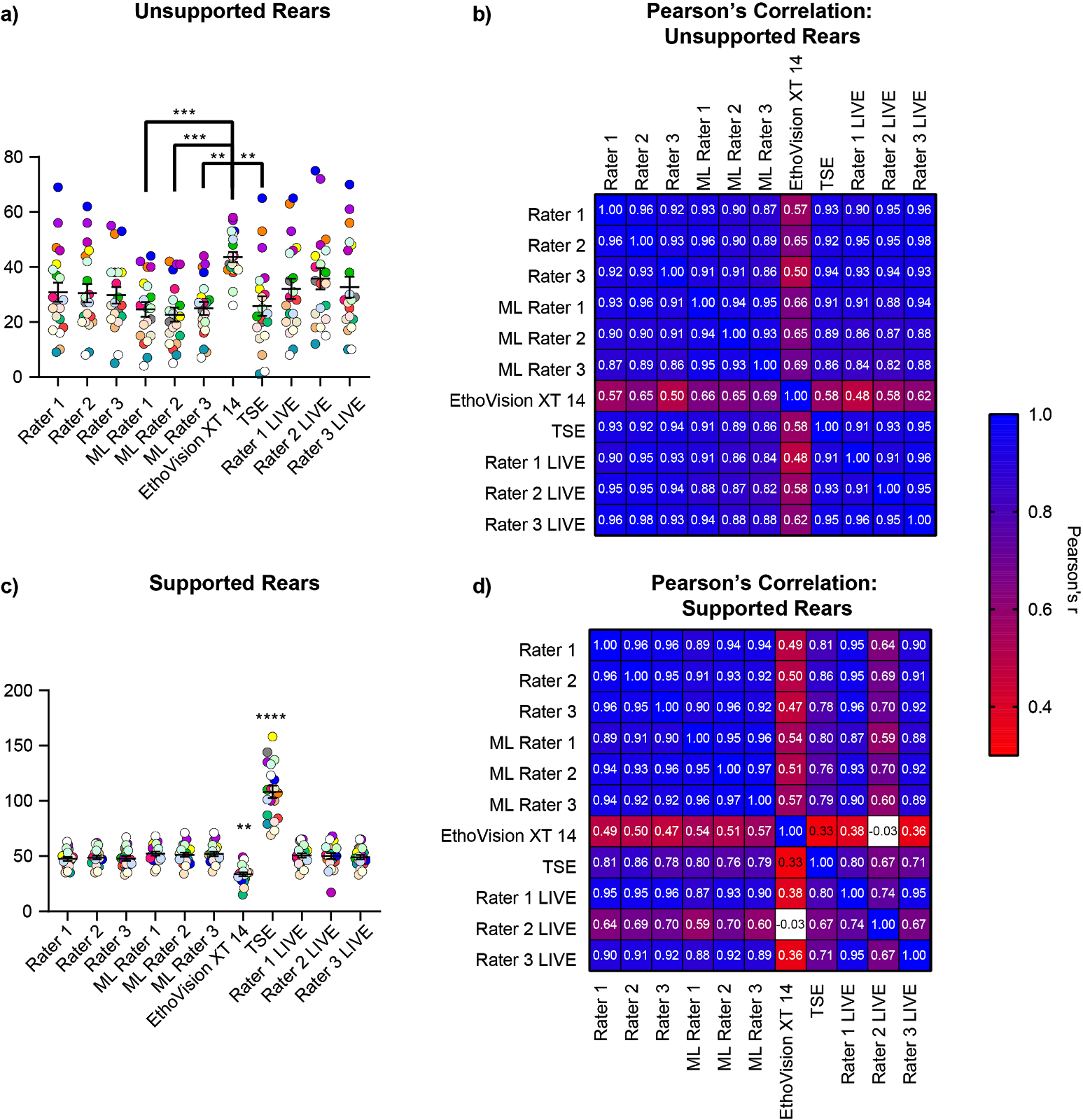
**Figure S10**. **An expanded version of Figure 5.** Absolute values of supported and unsupported rears in the OFT, and correlation plots to compare different scoring techniques. **(a,c)** The number of unsupported **(a)** and supported **(c)** rears reported by the three human annotators (rater 1-3), the three machine learning classifiers generated using the training data from the human annotators (ML rater 1-3), EthoVision XT14, the TSE Multi Conditioning system (TSE), and additionally the live scoring from the human annotators. **(b,d)** Correlation analysis comparing different scoring techniques. All raters and ML raters were significantly different to both EthoVision XT14 and TSE, only the highest p-values are reported for clarity. Colors represent individual animals and are consistent across analysis techniques for comparison (n=20), **=p>0.01, ***=p<0.001, ****=p>0.0001.


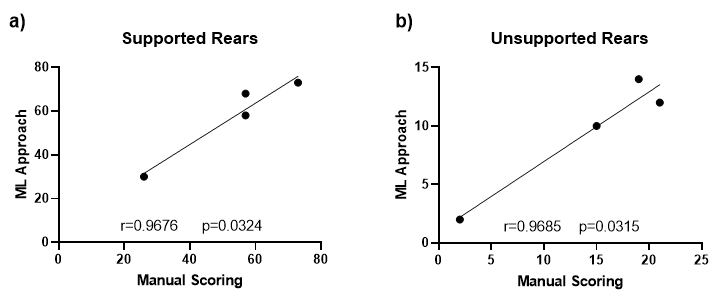


**S11. Automated scoring of rearing behavior in female mice.** The machine learning classifier, which was trained on male mice, accurately detects rearing in female mice. The automatically scored behaviors correlate highly with manual scoring, for both **(a)** supported rears and **(b)** unsupported rears.
