# Supplementary material for: Deep learning-based behavioral analysis reaches human accuracy and is capable of outperforming commercial solutions
Source: Neuropsychopharmacology. 2020 Jul 25;45(11):1942–52. doi: 10.1038/s41386-020-0776-y (PMC7608249; doi:10.1038/s41386-020-0776-y)
Supplement: Supplementary file 2 — Supplementary Material and Methods [file 41386_2020_776_MOESM2_ESM.docx]

**SUPPLEMENTARY MATERIAL AND METHODS**

**Deep learning based behavioral analysis reaches human accuracy and is capable of outperforming commercial solutions**

**Oliver Sturman^1,2^, Lukas von Ziegler^1,2^, Christa Schläppi^1,2^, Furkan Akyol^1,2^, Mattia Privitera^1,2^, Daria Slominski^1,2^, Christina Grimm^2,3^, Laetitia Thieren^2,4^, Valerio Zerbi^2,3^, Benjamin Grewe^2,5,6^, Johannes Bohacek^1,2^**

^1^ Laboratory of Molecular and Behavioral Neuroscience, Institute for Neuroscience, Department of Health Sciences and Technology, ETH Zurich, Switzerland

^2^ Neuroscience Center Zurich, ETH Zurich and University of Zurich, Switzerland

^3^ Neural Control of Movement Lab, Department of Health Sciences and Technology, ETH Zürich, Switzerland

^4^ Experimental Imaging and Neuroenergetics, Institute of Pharmacology and Toxicology, University of Zurich, Switzerland

^5^ Institute of Neuroinformatics, University of Zurich and ETH Zurich, Switzerland

^6^ Department of Information Technology and Electrical Engineering, ETH Zurich, Switzerland

**Animals**

C57BL/6J (C57BL/6JRj) mice (male, 2.5 months of age) were obtained from Janvier (France). Mice were maintained in a temperature- and humidity-controlled facility on a 12 hour reversed light–dark cycle (lights on at 08:15 am) in individually ventilated cages (SealSafe PLUS, Tecniplast, Germany) with food (M/R Haltung Extrudat, Provimi Kliba SA, Switzerland, Cat.# 3436) and water ad libitum. Cages contained wood chip bedding (LIGNOCEL SELECT, J. Rettenmaier & Söhne, Germany) nesting material (tissue paper) and a transparent red plastic shelter. Mice were housed in groups of 5 per-cage and used for experiments when 2.5-4 months old. All mice were given a minimum of 2 weeks to acclimatize to the light cycle and environmental conditions before testing. For each experiment, mice of the same age were used in all experimental groups to rule out confounding effects of age. All tests were conducted during the animals’ active (dark) phase from 12-5 pm. Mice were single housed 24 hours before behavioral testing in order to standardize their environment and avoid disturbing cagemates during testing [[43,44]](https://paperpile.com/c/Cj53Kb/mozC+bPNL). All procedures were carried out in accordance to Swiss cantonal regulations for animal experimentation and were approved under license 155/2015.

**Open Field Test (OFT)**

Open-field testing took place inside sound insulated, ventilated multi-conditioning chambers (TSE Systems Ltd, Germany). The open field arena (45 cm x 45 cm x 40 cm [L x W x H]) consisted of four transparent Plexiglas walls and a light grey PVC floor. Animals were tested under four equally spaced yellow lights (4 lux across the floor of the open field) with 65 dB of white noise playing through the speakers of each box. An infrared light illuminated the boxes so that an infrared camera could be used to record the tests. Prior to testing each animal, the entire open field arena was cleaned using 10 ml/l detergent (For, Dr. Schnell AG). The room housing the multi-conditioning chambers was illuminated with red LED lights (637 nm). Animals were removed from their homecage by the tail and placed directly into the center of the open field. The doors of the conditioning chamber were then swiftly closed. Tracking/recording was initiated by the Multi Conditioning System upon first locomotion grid beam break, whereas videos of this test were analyzed from the time the doors of the box were closed (approx. 3-5 s after first beam break). All open field tests were 10 minutes in duration. Distance, time in center, supported rears and unsupported rears were recorded in the OFT.

**Elevated Plus Maze (EPM)**

The elevated plus maze was made from grey PVC, with arms measuring 65.5 cm x 5.5 cm (L x W), elevated 61.5 cm. Prior to testing each animal, the entire elevated plus maze was cleaned using 70% EtOH in H₂O. The room housing the elevated plus maze was lit with two small lamps attached to the ceiling, they were adjusted until the open arms were at approximately 19-21 lux. A blackout curtain separated the room so that light from the screen would not alter the light conditions in the room and so the rater could not be seen by the animal. Animals were removed from their homecage by the tail and placed directly into the center of the EPM using a small starting box. Tracking/recording was initiated automatically by EthoVision XT14 (upon start condition: center point in arena for 2 seconds) and at the beginning of the video in DeepLabCut. All elevated plus maze tests were 10 minutes in duration. Distance, velocity, time in zone (open/closed arms + center) and head dips were recorded in the EPM.

**Forced Swim Test (FST)**

Animals were moved from the colony room to a holding room before immediate forced swim testing in 17.9-18.1°C water for 6 minutes. The forced swim took place in a plastic beaker (20 cm diameter, 25 cm deep, filled to 17 cm so no mouse could touch the bottom of the container with its tail, or escape). Tracking/recording was automatically initiated by EthoVision XT14 as the mouse made contact with the water. The beaker was cleaned and the water was changed and shortly before each swim. Overhead red LED lights (637 nm, invisible to the mice) dimly illuminated both the holding and testing rooms. Infrared LED strips illuminated a white POM-C  box onto which the beaker was placed. Distance, velocity and floating were recorded in the FST.

**3-Chamber Sociability Test (3CST)**

Animals were moved from the colony room to a holding room before immediate testing in the 3-chamber social interaction chamber (220lux) (Social Box (Panlab), Harvard Apparatus) where they were given the choice between investigating a novel object or mouse. Tracking/recording was automatically initiated by EthoVision XT14, the videos were then analysed using DLC. The arena was cleaned shortly before each test. Distance, velocity and time investigating the novel object/mouse were recorded.

**Barnes Maze**

Animals were moved from the colony room to a holding room before immediate testing on the Barnes Maze (105cm diameter, featuring 22 (5cm diameter) equally spaced holes, 260 lux). Tracking/recording was initiated shortly before the animal was released from a start box in the center of the table. The arena was cleaned shortly before each test. Distance, velocity and latency to the target hole were recorded.

**Optogenetic targeting of striatal D2 medium spiny neurons**

Three adult female Adora2a-Cre mice (aged 8-12 weeks at the start of experimental procedures) were used (Gong *et al.*, 2007) and kept in standard housing on a regular 12 h light/dark cycle. To elicit subtle ipsiversive (i.e. clockwise) rotation in mice as demonstrated previously (Hintiryan *et al.*, 2016; Kravitz *et al.*, 2010; Lee *et al.*, 2016), we optogenetically targeted D2-expressing medium spiny neurons in the right ventrolateral sub-region of the caudate putamen. Mice were injected unilaterally (AP 0.48mm; ML 1.5 mm; DV 3.0 mm, relative to bregma) with 600 nl of viral construct (AAV5-hEF1α-dlox-hChR2(H134R)-EYFP(rev)-dlox-WPRE-hGHp, and an optical fiber (400 μm, NA=0.66) was placed 0.2 mm above the injection site. After 3-4 weeks of recovery, animals were habituated to the testing room for 45 minutes, then connected to the optic fiber and placed into the open-field arena (40 cm x 40 cm x 40cm, **≈**165 lux) for 15 minutes. Following this 15-min habituation period, animals were subjected to five stimulation blocks: three minutes baseline, followed by 20 seconds of 20 Hz stimulation and 40 seconds rest (x5). The stimulus was delivered via a Fiber Optic Rotary Joint Patch Cable (400 μm, NA=0.39; ThorLabs, Inc) connected to a DPSS laser (CNI laser; wavelength 488 nm, 20 Hz, 7 mW).

**Noldus EthoVision**

EthoVision XT14 was used to acquire all forced swim and elevated plus maze videos and to analyze all of the open field videos. The automatic animal detection settings were used for all tests, slight tuning of these settings was performed using the fine-tuning slider in the automated animal detection settings to ensure the animals could be tracked throughout the entire arena. We ensured there was a smooth tracking curve and that the center point of the animal remained stable before analysis took place.

**DeepLabCut (DLC)**

DeepLabCut 2.0.7 was used to track all points of interest **(Figure 1)**. The OFT network was trained using 15 frames from 8 randomly selected videos for 1030000 iterations (multistep: 0.005 (10000 iterations), multistep: 0.02 (430000 iterations), multistep: 0.002 (730000 iterations), multistep: 0.001(1030000 iterations). The EPM network was trained using 10 frames from 13 randomly selected videos for 250000 iterations (multistep: 0.005 (2500 iterations), multistep: 0.02 (12500 iterations), multistep: 0.002 (187500 iterations), multistep: 0.001(250000 iterations). 10 outlier frames from each of the training videos were then corrected, with points with a p < 0.7 being relabeled. The network was then refined using the same number of iterations. The FST network was trained using 20 frames from 28 randomly selected videos for 250000 iterations (multistep: 0.005 (20000 iterations), multistep: 0.02 (100000 iterations), multistep: 0.002 (175000 iterations), multistep: 0.001(250000 iterations). 20 outlier frames from each of the training videos were then corrected, for which points with a p < 0.7 were relabeled. The network was then refined using the same number of iterations).The 3-Chamber Social Interaction network was trained using 20 frames from 5 randomly selected videos for 1030000 iterations (multistep: 0.005 (10000 iterations), multistep: 0.02 (430000 iterations), multistep: 0.002 (730000 iterations), multistep: 0.001(1030000 iterations). The Barnes Maze network was trained using 20 frames from 13 randomly selected videos for 250000 iterations (multistep: 0.005 (2500 iterations), multistep: 0.02 (12500 iterations), multistep: 0.002 (187500 iterations), multistep: 0.001(250000 iterations). The data generated by DeepLabCut was then processed using custom R Scripts that are available in the supplementary data (<https://github.com/ETHZ-INS/DLCAnalyzer>).

**TSE Multi Conditioning System**

Locomotion was tracked using an infrared beam grid; an additional beam grid was raised 6.5 cm above the locomotion grid to measure rearing. The central 50% (1012.5 cm^2^) was defined as the center of the arena. To automatically distinguish supported from unsupported rears, we empirically determined the area in which mice could not perform a supported rear. Thus, all rears within 12.5 cm of the walls were considered supported rears, while rears in the rest of the field were considered unsupported rears. Rearing was defined as an interruption of a beam in the z-axis for a minimum of 150 ms. If another rear was reported within 150 ms of the initial rear, it was counted as part of the initial rear.

**Analysis of DLC Coordinates**

X and Y coordinates of tracked points as determined with DLC, were imported into R Studio (v 3.6.1) and processed with custom scripts (https://github.com/ETHZ-INS/DLCAnalyzer). Values of points with low likelihood (> 0.95) were removed and interpolated using the R package “imputeTS” (v 2.7). The speed and acceleration of each point was determined by integrating the animal's position over time. Points of interest relating to the arenas were tracked and median xy-coordinates were used to define the arenas *in silico*. The pixel-to-cm conversion ratio for each video was determined by comparing the volume of the arena *in-silico* in px^2^ to the measured size of the arena in cm^2^. Zones of interest were calculated from the arena definitions using polygon-scaling functions. We defined 6 zones in the OFT: the center (scaling factor = 0.5), the periphery (scaling factor = 0.8) and the 4 corners (scaling factor = 0.2, centered on the corners); and 3 zones in the EPM (open arms, closed arms and center). Integration of the body center over the entire video was used to calculate metrics such as total distance, average speed and time in zone for each mouse. Further, a speed cutoff (5 cm/s) was set to determine when and how long an animal was moving and its average speed whilst moving. Time floating in the FST was determined by analyzing the rate of change of the polygon area formed by joining the head-center, tailbase, bcl and bcr **(Figure 1a and Figure 4b)**. Whenever this rate of change (smoothed with a rolling mean over ±5 frames) was below a preset cutoff (15 px^2^/frame) the animal was considered floating. Head dips in the EPM were scored by observing if the ‘head center’ point was outside of the EPM arena. Additionally, to prevent rears in the closed arm from registering as head dips only observations in which the ‘neck’ was not in any closed arm were considered.

Head angle was calculated by first creating two vectors, a = (nose – neck) and b = (neck – tailbase) and calculating the absolute angle with the following formula. Angle = arctan2(det, dot), where det is the determinant (ax*by – bx * ay) and dot the dot product (ax * bx + ay * by). This analysis was done for each frame. We then used the average of this readout within each bin to create a single value for the average head angle.

For the Barnes maze, we trained a network that tracked the center of each hole (total of 22 holes). The median x and y values of an entire video were used as final hole center coordinates. Then, hole visits were determined by checking if distance (nose – holecenter) < hole radius for each frame. Time to first visit was determined by the first frame at which this was true, time spent at each hole was determined by the number of frame for which this was true. For the 3-chamber sociability test we used a similar approach, where the center of both round objects was tracked and the interaction recorded whenever the distance between the nose and center of an object was bellow a cutoff value.

**Features for floating detection**

Features for floating detection were created from acceleration data of individual points. All mouse points were included except the tailtip and tailcenter. Acceleration was calculated by first integrating position over time to resolve velocity and then integrating velocity over time. Absolute values of acceleration were used for features. This resulted in 11 acceleration measures at each frame. To represent temporal information for each frame the information of the +-20 frames centered around it were used. This (11 x 41) acceleration sequence of 11 points was flattened into a single 451-dimensional array that was used as input for the neural network.

**Time Resolved Skeleton Representation**

A position and orientation invariant skeletal representation was created from the DLC tracked coordinates at each frame. The distances between pre-determined tracking point pairs, angles between pre-determined vector pairs and the areas of pre-determined polygons in each frame were calculated. The resulting skeletal representation contained a total of 10 distances, 6 angles and 4 areas. Additionally, two Boolean variables were included to check if the points (in this case nose and head center) were inside the arena or not. These resulting 22 variables were used as features for the supervised machine learning.

To further incorporate temporal information, the skeletal representation was expanded to create short sequences over a predetermined time interval. In this case, an interval of ±15 frames was chosen. The skeletal data from each frame was then flattened into a longer skeleton-sequence-array that describes a short sequence of the skeleton of ± 15 frames for each frame centered on the frame. Therefore, the resulting input dimension was 682 (31 x 22). Flattening was done sequentially, where first the 22 features of the N-15th frame and last the 22 features of the N+15th frame were added (final array structure: 22 features of N-15th frame, 22 features of N-14th frame, … , 22 features of Nth frame, …, 22 features of N+15th frame). The classification of this 682-dimensional array was used for the Nth frame. The first and last 15 frames of each video that did not allow for such an expansion were omitted.

**Machine Learning Approach**

In order to create a training data set, 20 videos of the OFT were manually labeled (using VIA video annotator [[45]](https://paperpile.com/c/Cj53Kb/5cS6)), indicating the onset and offset of selected behaviors. Labeled behaviors include ‘supported rear’, ‘unsupported rear’, and by default ‘none’. Videos were labeled by three independent raters. These sets of labeling data were used to train multiple neuronal networks for the classification of the selected behaviors (labelling data can be accessed here: <https://github.com/ETHZ-INS/DLCAnalyzer/data>. All videos can be found here: <https://zenodo.org/record/3608658>). For each labeled video the other 19 videos were used to train a feed forward neural network for classification. The model was then cross-validated on the single video not included in the training set. This process was repeated with each raters’ labeling data, resulting in a total of 60 models and cross validations. The R package for tensorflow and keras were used for machine learning. Training and testing data were normalized within videos using a Z-score (x – mean(x) / sd(x)) method on non-boolean parameters. The training data was randomly shuffled before training. A sequential model with two hidden layers was trained (input shape: N = 682, L1: dense layer, N = 256, dropout rate = 0.4, activation = ‘relu’; L2: dense layer, N = 128, dropout rate = 0.3, activation = ‘relu’; and an output layer with: 4 nodes, activation = ‘softmax’). The network was trained for 10 epochs with a batch size of 32. The optimizer ‘rmsprop’, the loss function ‘categorical_crossentropy’ and metric ‘accuracy’ were used. Accuracy on the cross validation set was determined on a frame to frame basis. However, to remove single frame misclassifications the final classification was integrated over a period of ±5 frames.

**Computer Specifications and Prior Experience of Experimenters**

We used a Dell XPS 8930 workstation (Intel Core i7-8700K, 16GB RAM(DDR4), 512GB SSD, 2TB HDD, Nvidia GTX 1080 GPU, to implement the DLC-based approach, and to train the machine learning classifiers.  We investigated the labelling, training and analysis times of networks that use different numbers of labelled points. It takes an experienced experimenter approximately 5 minutes to label 20 frames with 18 points of interest (13 labels on the mouse and 4 or more labels on the arena, based on its complexity). Using the same computer described above, the network then trains overnight (ca. 11hrs) and a 10-minute video is analyzed in ca. 9 minutes (see Supplementary Table S1).

**Behavior analysis**

All annotators were trained by an expert behaviorist and reached a consensus on what constitutes each behavior before scoring any behavior. In the case of large discrepancies between annotators the annotator in question was retrained, re-blinded and given the opportunity to score again. This was not the case for the live scoring, where the annotators initial values were reported. Floating was classified as the lack of directed movement of the animal in the swimming arena, however very minor movements of the animal attributed to balance were also classified as floating. Supported rearing was classified as the movement of the animal onto its hind limbs and elongation of the body with some form of contact with the walls of the arena, whereas unsupported rearing was classed as the movement of the animal onto its hindlimbs and elongation of the body with no contact with the walls of the arena. Head dips in the EPM were defined as the movement of a mouse's head over the arm of the plus maze with accompanying curvature of the neck that would imply the animal was looking at the floor. Experienced raters were able to score a 10-minute behavior video in approximately 40-60 minutes, depending on the number of events that had to be scored.

**Statistical Analysis**

Data was tested for normality and all comparisons between normally distributed datasets containing two independent groups were performed using unpaired t-tests (2-tailed), whereas all comparisons between more than two groups were performed using one-way ANOVAs in order to identify group effects. Significant main effects were then followed up with post hoc tests (Tukey's multiple comparison test). We also report the Coefficient of Variation (CV) in order to show the dispersion of the data around the mean.
